# Porcine Respiratory Coronavirus as a Model for Acute Respiratory Coronavirus Disease

**DOI:** 10.3389/fimmu.2022.867707

**Published:** 2022-03-28

**Authors:** Sarah Keep, Brigid Veronica Carr, Fabian Z. X. Lean, Albert Fones, Joseph Newman, Giulia Dowgier, Graham Freimanis, Eleni Vatzia, Noemi Polo, Holly Everest, Isobel Webb, Adam Mcnee, Basu Paudyal, Nazia Thakur, Alejandro Nunez, Ronan MacLoughlin, Helena Maier, John Hammond, Dalan Bailey, Ryan Waters, Bryan Charleston, Toby Tuthill, Paul Britton, Erica Bickerton, Elma Tchilian

**Affiliations:** ^1^The Pirbright Institute, Pirbright, United Kingdom; ^2^Department of Pathology, Animal and Plant Health Agency, Addlestone, United Kingdom; ^3^Research and Development, Science and Emerging Technologies, Aerogen, Galway, Ireland

**Keywords:** porcine respiratory coronavirus, lung pathology, swine, SARS-CoV-2 model, tracheal organ cultures

## Abstract

In the light of the severe acute respiratory syndrome coronavirus 2 (SARS-CoV-2) pandemic, we have developed a porcine respiratory coronavirus (PRCV) model for in depth mechanistic evaluation of the pathogenesis, virology and immune responses of this important family of viruses. Pigs are a large animal with similar physiology and immunology to humans and are a natural host for PRCV. Four PRCV strains were investigated and shown to induce different degrees of lung pathology. Importantly, although all four strains replicated equally well in porcine cell lines *in vitro* and in the upper respiratory tract *in vivo*, PRCV strains causing more severe lung pathology were also able to replicate in *ex vivo* tracheal organ cultures as well as *in vivo* in the trachea and lung. The time course of infection of PRCV 135, which caused the most severe pulmonary pathology, was investigated. Virus was shed from the upper respiratory tract until day 10 post infection, with infection of the respiratory mucosa, as well as olfactory and sustentacular cells, providing an excellent model to study upper respiratory tract disease in addition to the commonly known lower respiratory tract disease from PRCV. Infected animals made antibody and T cell responses that cross reacted with the four PRCV strains and Transmissible Gastroenteritis Virus. The antibody response was reproduced *in vitro* in organ cultures. Comparison of mechanisms of infection and immune control in pigs infected with PRCVs of differing pathogenicity with human data from SARS-CoV-2 infection and from our *in vitro* organ cultures, will enable key events in coronavirus infection and disease pathogenesis to be identified.

## Introduction

Respiratory viruses are a major threat to global health, with coronaviruses (CoVs) being responsible for three major human epidemics since 2003, including the ongoing Covid-19 pandemic caused by the severe acute respiratory syndrome coronavirus 2 (SARS-CoV-2). In each case, CoV emergence in humans has been associated with zoonotic transmissions from animals. Many livestock species including pigs are infected with CoVs with variable morbidity and mortality associated with lung disease and immunological impairment, which may lead to substantial economic losses. Porcine respiratory CoV (PRCV) are globally endemic in pigs and with the emergence of porcine epidemic diarrhoea virus (PEDV) and the detection of porcine delta coronavirus (PDCoV) in humans, better understanding of the fundamental biology and disease mechanisms of these CoVs is essential (1, 2).

PRCV was first reported in Belgium in the early 1980’s and subsequently found to be a respiratory variant of the *alphacoronavirus* Transmissible Gastroenteritis Virus (TGEV) (3). TGEV, was described in 1946 causing enteric disease characterised by acute diarrhoea, vomiting and dehydration with high mortality in neonatal piglets (4). In contrast PRCV infection is mild and often sub-clinical inducing mild broncho-interstitial pneumonia and neutrophil infiltration (5, 6). Since the emergence of PRCV, the impact of TGEV has been greatly reduced due to cross protective immunity (7). Investigation of the PRCV genome identified the presence of an approximately 600 nucleotide (nt) deletion within the 5’ end of the S gene, resulting the loss of at least 200 amino acids within the N-terminal region of the S protein accounting for the loss of enteric tissue tropism and altered disease presentation (8). PRCV is thought to be unable to bind to sialic acid, preventing infection of the gastroenteric tract (9, 10). The deletion, however, does not affect the ability to bind to the TGEV host receptor aminopeptidase N (APN) (11, 12). Additionally, PRCV contains deletions in accessory gene 3, resulting in the loss of both or either 3a and 3b proteins, which may affect *in vivo* tissue tropism and clinical disease (8, 13–15). The relationship between TGEV and PRCV is complex with several strains/variants of PRCV identified globally each containing different sized deletions in the S and 3a/b genes with potential to influence disease outcome (16).

Although the majority of PRCV infections are subclinical unless accompanied by secondary infections, pneumonia following acute PRCV infection could resemble those of severe acute respiratory syndrome (SARS) patients (5, 17). However, the lesions and severity vary depending on the PRCV strain (18). PRCV ISU-1 strain induced pathology was proposed as a model to evaluate the effect of the corticosteroid dexamethasone treatment on SARS-CoV-induced pneumonia. Dexamethasone alleviated the pneumonia during early infection but continued use exacerbated the later stages of infection (5). Less systemic pro-inflammatory cytokines were detected in pigs infected with PRCV compared to influenza virus, consistent with the usually mild clinical signs (6).

Currently mouse, ferret, hamster and non-human primate models have been developed to study SARS-CoV-2, but these are not natural host species for infection, and it is not clear which best recapitulate human disease (19, 20). In contrast the pig is a natural host for several porcine CoVs and is physiologically anatomically and immunologically similar to humans (21–23). As with SARS-CoV-2, PRCV infection can be mild or asymptomatic, but in some instances can lead to pneumonia. Better understanding of mechanisms that lead to mild or severe disease is essential for developing new control strategies for emerging severe coronavirus diseases of livestock or humans. However, little is known about the mechanisms of PRCV pathogenicity and protective immunity in pigs. Here we evaluated the *in vitro* growth characteristics and *in vivo* clinical, virological, pathological and immunological responses to four PRCV strains with differing pathogenicity in pigs, the natural host for PRCV. The long-term aim of this programme of work will be to understand the coronavirus genomic changes that result in enhanced disease and to understand local and systemic protective immune responses to respiratory coronaviruses.

## Materials and Methods

### Cells and Viruses

Swine testis (ST) cells were purchased from ATCC (ATCC CRL-1746) and were propagated using advanced minimum essential media (MEM; Gibco) supplemented with 5% foetal bovine serum (FBS), 2 mM L- glutamine and 10,000 units (U) per ml of penicillin and 10,000 µg/ml streptomycin (Life Technologies). LLC-PK1 cells, a porcine kidney epithelial cell line, were provided by the central services unit (CSU) at The Pirbright Institute and were propagated using 199 media (Sigma) supplemented with 10% FBS and 10,000 U/ml penicillin and 10,000 µg/ml streptomycin. HEK293T cells provided by CSU at The Pirbright Institute were maintained in Dulbecco’s modified Eagle medium (DMEM) supplemented with 10% FBS, 1% sodium pyruvate solution (Sigma-Aldrich) and 10,000U/ml of penicillin and 10,000 µg/ml streptomycin, from here called cDMEM-10. All cells were maintained at 37°C, 5% CO_2_.

PRCV strain AR310, abbreviated to 310, was purchased from ATCC (ATCC VR2384) and ISU-1 from Bei Resources (NR-43286). 310 was originally isolated from the small intestine of a pig within a TGEV endemically infected herd located in Arkansas, USA (24) ISU-1, GenBank accession number DQ811787.1, was isolated from nasal swabs collected from pigs in Indiana, USA (25). PRCV strains 86/137004 (abbreviated to 137) and 86/135308 (abbreviated to 135) were both isolated in 1986 from pigs in the UK and were gifts from S. Cartwright (26) (Animal and Plant Health Agency, APHA). Both 135 and 137 were isolated from infected respiratory tract tissue. The TGEV strain FS772/70 was a gift from S. Cartwright (27). All viruses were propagated on ST cells using PRCV medium (EMEM supplement with 0.02% yeast extract, 10% TPB, 2 mM L-glutamine, 10,000 U/ml penicillin and 10,000 µg/ml streptomycin) and quantified *via* titration, also in ST cells.

### Full Genome Sequencing of PRCV 135, 137, 310 and ISU-1

RNA was extracted from PRCV strains 135, 137, 310 and ISU-1 propagated on ST cells using the Qiagen viral RNA kit following the manufacturer’s instructions, including addition of carrier RNA. RNA quality was analysed using an RNA screentape on a Tapestation 4200 (Agilent, Santa Clara) and a Qubit RNA BR kit (Qiagen, Hilden). cDNA and library preparation were carried out in duplicate for each sample. Sequencing library preparation was performed with an input of 200 ng total RNA for each sample, using the NEBNext directional Ultra II RNA-Seq kit (NEB, Ipswich, MA). Library quality control was performed using the Tapestation DNA1000 kit and Qubit BR kit, prior to pooling. Sequencing pools were quantitated using the NEBNEXT Illumina library quantitation kit (NEB, Ipswich, MA) before dilution for sequencing. All libraries were run on an Illumina MiSeq, using a MiSeq reagent v3 600 cycle cartridge (Illumina, San Diego). The data generated were subjected to quality control checks on the raw fastq files using FastQC. Filtered reads for standard quality score threshold were aligned to the reference genome PRCV ISU1 (GenBank: DQ811787) using both BWA-MEM (version 0.7.12-r1039), and Bowtie2 (version 4.1.2) for comparison. A consensus sequence was generated for each sample and the duplicate samples compared. The consensuses generated by each duplicate were identical and therefore a single consensus was considered for further analysis and submitted to NCBI GenBank, accession numbers OM830318 (PRCV 135), OM830319 (PRCV 310), OM830320 (PRCV 137) and OM830321 (PRCV ISU-1). Sequence alignments of the S and 3b genes were performed with Snapgene, version 5.2.3 (**Supplementary Figures 1,2**).

### Plaque Assays in ST Cells

ST cells were seeded in 12 well sterile tissue culture plates and incubated until confluent. Ten-fold serial dilutions were prepared using PRCV medium. Cells were washed with phosphate buffer saline (PBS) prior to inoculation with 250 µl per well of virus in either duplicate or triplicate. After 1 h incubation at 37°C, the inoculum was removed and 2 ml N,N-Bis(2-hydroxyethyl)-2aminoethanesulphonic acid) medium containing 1% agar (suitable for cell culture; Sigma) was added. Plaques were visualized 96 hpi using 0.1% crystal violet (H_2_O) following fixation with 3.3% formaldehyde (PBS), and the number of plaque forming units (PFU) per ml calculated.

### Growth Kinetic Assays

ST or LLC-PK1 cells were seeded in 6 well sterile tissue culture plates and incubated until confluent. Cells were washed twice with PBS prior to infection with 10^4^ PFU of either PRCV or TGEV strains in 500 µl PRCV medium. Mock infected cells were inoculated with medium only. Inoculated cells were incubated for 1 h, 37°C after which the inoculum was removed, the cells washed once with PBS, and 3 ml PRCV medium added per well. At defined intervals supernatant was harvested and titrated in either duplicate or triplicate in ST cells to determine the quantity of infectious progeny. RNA was extracted from the infected cells using RNeasy mini kit (Qiagen) including an on column DNAse treatment; viral RNA load was determined by qRT-PCR.

### Analysis of RNA Synthesis by Quantitative Real-Time PCR (qRT-PCR)

Reverse transcription, using random primer, of 500 ng total RNA (cell lystate), 400 ng (BAL derived RNA), 500 ng (Nasal or trachea swab RNA) was performed using the Superscript IV. Reverse Transcriptase kit (Invitrogen, Waltham, MA, USA) as per the manufacturer’s protocol. Viral RNA load was quantified using quantitative real-time PCR (qRT-PCR) using primers and probes targeting the N gene as follows: forward primer 5’-ATAGACAAACTCGCTATCGC-3’, reverse primer 5’-CAACCCAGACAACTCCATC-3’, and probe FAM-TAGGCACTGGACCTCATGCA-TAMRA. Quantitative RT-PCR was performed with FastAdvance Mastermix (Applied Biosystems) using 500 nM forward and reverse primers, 300 nM probe and 100 ng of cDNA as normalized input. An applied Biosystems QuantStudio/7500 Fast real-time PCR instrument (Life Technologies) was used with the following cycling conditions: 10 min at 95°C, 35 cycles of 10 s at 95°C, 10 s at 60°C and 10 s at 75°C. Standard curves were generated to allow quantitation of RNA copy numbers based on cDNA levels using a pCR4-TOPO (Invitrogen) plasmid containing the sequence amplified by the set of primers listed above. The resulting Ct results were used to calculate the log of relative RNA copies using the linear equation from the standard curve.

### Infection of Primary Tracheal Organ Cultures

Tracheal organ cultures (TOCs) were prepared from trachea extracted from a 4-week-old piglet. The trachea was washed with PBS and excess fat removed using a scalpel. The trachea was sectioned into rings of approximately 3 mm, and each ring placed in a glass test tube (10 mm by 16 mm) with 1 ml of medium (EMEM supplemented with 40 mM Hepes, 2 mM L-glutamine, 10,000 U/ml penicillin and 10,000 µg/ml streptomycin and 10,000 U/ml nystatin). The TOCs were incubated for 48 h prior to infection, in a non-CO_2_ incubator (Incudrive-S, Life Technologies) at 37°C, 7 – 8 revolutions per h. Each TOC was inoculated with 500 µl PRCV medium only or 500 µl PRCV medium containing 1 x 10^4^ or 1 x 10^5^ PFU of either PRCV strains 135, 137, ISU-1, 310 or TGEV 772/70. TOCs were incubated for 1 h at 37°C, no rotation, after which the inoculum was removed and replaced with 1 ml 1 x PRCV media. TOCs were incubated for a further 23 h at 37°C, 7 - 8 rev per h. At 24 hpi the supernatant was harvested and the quantity of infectious virus determined by titration in ST cells. TOCs were either discarded or fixed for investigation by confocal microscopy.

### Confocal of *Ex Vivo* Tracheal Organ Cultures

Whole *ex vivo* TOCs were fixed in 4% formalin for 72 h, washed in PBS and frozen at -80°C in optimal cutting temperature compound (OCT compound). Frozen TOCs were sectioned using a cryostat (Leica Cryostat CM1860 UV) into 100 µm thick transverse sections. Individual sections were placed onto glass slides and allowed to air dry for 30 min before residual OCT compound was removed. Tissue sections were permeabilized with 0.1% Triton X-100 for 20 min and blocked with 0.5% PBS/BSA for 1 h. Tissue sections were labelled for 1 h with either the mouse monoclonal antibody, DA3, against the N protein of TGEV FS772/70 strain which targets the 23 terminal residues of the nucleoprotein (N) protein (28) or anti-dsRNA (Absolute Antibody, Ab00458-23.0) or co-labelled with anti-N (DA3) and anti-dsRNA together. The antibodies were diluted 1:400 and 1:1000 respectively in 0.5% BSA. Labelled tissues sections were washed three times in PBS prior to secondary antibody labelling for 1 h. Secondary antibodies were Alexa Fluor^®^ 488 Goat anti-mouse IgG2a (Invitrogen, A-21131) for the detection of anti-N and Alexa Fluor^®^ 568 Goat anti-Rabbit IgG (H+L) (Abcam, ab175471) for the detection of anti-dsRNA. Both were diluted 1:1000 in PBS/BSA. After three washes, nuclei were stained for 10 min with 4’,6-diamidino-2-phenylindole (DAPI) diluted 1:10,000 in deionised water. Tissue sections were washed with deionised water. GeneFrames, ~ 65 µl depth (1.5 x 1.6 cm - Thermo Scientific™) were placed around each labelled tracheal section and Vectashield mounting medium added followed by a coverslip. Tissue sections were imaged on a Leica confocal microscope.

### *In Vivo* PRCV Challenge

All experiments were approved by the ethical review processes at the Pirbright Institute and Animal and Plant Health Agency and conducted according to the U.K. Government Animal (Scientific Procedures) Act 1986 under project license PP7764821. Both institutes conform to Animal Research: Reporting of *In Vivo* Experiments guidelines. Prior to infection, all pigs were screened for the absence of PRCV antibodies using the INgezim Corona Diferencial ELISA (Ingenasa, Spain). Clinical parameters including demeanour, appetite, respiratory signs, sneezing, coughing, nasal and eye discharge, faeces consistency and rectal temperature were assessed.

#### Pilot Study to Examine the Virological and Pathological Characteristics of PRCV 135, 137 ISU-1 and 310 Strains

A high health farm with stringent biosecurity was identified as the source of pigs for this study by screening archival serum samples, taken for routine health surveillance, for PRCV antibodies. This indicated there was no circulation of PRCV. From this farm a total of eight Landrace/Yorkshire cross gilts of 6 to 10 weeks of age (23.5 kg average weight) were randomly divided into 4 treatment groups. Two pigs per group were challenged intranasally and intra-tracheally with 2 x 10^7^ PFU (in 5 ml RPMI, 25mM Hepes) of either 135, 137, ISU-1 or 310. Intra-nasal delivery (1 ml per nostril) was performed using a mucosal atomisation device (MAD^®^ Nasal, Wolfe Tory Medical Inc., Salt Lake City, US) and 3 ml were delivered trans-tracheally. All pigs were culled by overdose of anaesthesia 5 days post infection (DPI). Nasal swabs were collected immediately prior to inoculation, referred to as day 0, and each day post inoculation and stored in 1 ml of Trizol (Life Technologies) for analysis of viral RNA load by qRT-PCR or 1 ml virus transport media (tissue culture medium 199 (Sigma-Aldrich, UK) supplemented with 25 mM Hepes, 0.035% sodium bicarbonate, 0.5% BSA, penicillin, streptomycin and nystatin) for analysis of infectious viral load by titration in ST cells. Deep nasal and tracheal swabs were also collected at post-mortem. Whole blood, nasal turbinates, trachea, tracheobronchial lymph nodes (TBLN), bronchial alveolar lavage (BAL), lung, spleen, retro-pharyngeal and mesenteric lymph nodes were collected at post-mortem and processed as previously described (29, 30).

#### *In Vivo* Challenge With PRCV 135 Strain

Eighteen Landrace/Yorkshire cross gilts (16 kg average weight), from the same farm as in the first pilot study above, were randomly divided into two groups. Nine pigs were inoculated with 1 x 10^7^ PFU (in 5 ml RPMI, 25 mM Hepes) of 135 strain intranasally using a MAD and trans-tracheally as above. Aerosol (Aer) administration was performed using a small droplet size, vibrating mesh nebulizer (Aerogen Solo; Aerogen, Galway, Ireland) attached to a custom-made veterinary mask (29). For Aer delivery, 2 ml of 135 strain containing 1 x 10^7^ PFU was administered over 6–10 min following sedation. Three pigs per group were culled by an overdose of anaesthesia at 4, 9 and 16 DPI and the same tissues harvested as in the first pilot study.

### Determination of Infectious Virus in Tissues

Tissues were homogenised in 1 ml PBS containing 10,000 U/ml penicillin and 10,000 µg/ml streptomycin using 5 mm stainless steel beads and a Tissue Lyser II (Qiagen). 0.5 g of lung accessory lobe, 0.5 g of tracheal tissue, 0.5 g (from the pilot study) and 0.1 g of nasal turbinates were homogenised. The resulting supernatant was clarified by low-speed centrifugation. The quantity of infectious virus was determined by titration in ST cells.

### Gross Pathology

Lungs harvested at necropsy were assessed blindly by a pathologist. Both dorsal and ventral surfaces were assessed for reddening and consolidation of lung lobes as previously described (31). Lung images taken at necropsy were analysed using Image J (Version 1.52a) and assessed blindly by a different operator to estimate percentage of lung lesions.

### Histology and Immunohistochemistry

Formalin fixed tissues were processed by routine histology method. Hematoxylin and eosin staining and immunohistochemistry (IHC) against PRCV nucleoprotein (N) were performed on serially sectioned formalin-fixed paraffin-embedded tissues. Briefly, 4 μm sections were dewaxed and dehydrated through xylene and graded alcohol, endogenous peroxidase activity quenched with a methanol and hydrogen peroxide, followed by unmasking of epitope with heat-mediated antigen retrieval in pH 9 buffer at 100°C for 10 mins (Dako). Slides were assembled into Shandon Sequenza cover plates to facilitate IHC (Shandon, USA). Tissue sections were incubated with an anti-N mouse monoclonal antibody DA3 at 0.1 µg/ml or concentration matched isotype mouse monoclonal antibody for 1 hr at RT and then with Dako ENVISION™ polymer for 20 min at RT. Immunolabelling was performed at room temperature and sections were washed three times with Tris-buffered saline between incubations. Immunolabelling was visualized using 3,3-diaminobenzidine (Sigma Aldrich) and counterstained in Mayer’s haematoxylin (Surgipath, UK). Slides were dehydrated in absolute alcohol, cleared in xylene and mounted using Dibutyl Phthalate Xylene and glass coverslips. A serial section was stained with haematoxylin and eosin for histopathology evaluation. Positive control slides containing either TGEV or PRCV infected or uninfected cells prepared in agarose matrix were included during IHC experiment to confirm immunolabelling (32).

### Expression of Full Length PRCV ISU-1 Spike Protein

The spike sequence from PRCV ISU-1 (GenBank Accession number: DQ811787.1) was modified to replace the coding region for the C-terminal transmembrane and cytoplasmic domains, with a T4 foldon trimerization domain followed by a His^8^ tag. The sequence coding for amino acids E914 and L915 was mutated to two prolines in an attempt to stabilise the spike in a pre-fusion state similar to that described for other coronavirus spikes. The sequence was optimised to human codons and synthesised into the plasmid expression vector pTwist CMV BetaGlobin, to create vector pPRCV-ISU1-PP-Foldon. The spike was expressed in Expi293F cells (ThermoFisher) according to the manufacturer’s instructions. Culture supernatants were clarified by centrifugation and purified through 5 mL HisTrap FF column (GE Healthcare). Fractions containing spike were concentrated and the excess imidazole removed by using buffer exchange columns (Zeba, Merck).

### Generation Lentiviral-Based PRCV Pseudo Particles

The spike sequence from PRCV ISU-1 was optimised to human codons and synthesised in plasmid expression vector pTwist CMV BetaGlobin (Twist Bioscience), to create vector pPRCV-ISU1-PP. HEK293T cells were plated at 4 x 10^5^ cells per 6-well plate in cDMEM-10. The following day, the cells were transfected with 0.5 µg of pPRCV-ISU1-PP, along with 0.6 µg p8.91 (encoding HIV-1 gag-pol) and 0.6 µg CSFLW (the firefly luciferase reporter-expressing lentivirus-backbone) using 10 µL polyethylene imine (PEI; Sigma-Aldrich). Transfection mixtures were removed the next day and replaced with 3 ml cDMEM-10. Cells were incubated for 48 h with two harvests of supernatant taken at 24 and 48 h which were pooled, centrifuged at 1300 x g for 10 minutes at 4°C to remove cellular debris and then aliquoted and frozen at -80°C.

The sequence for porcine APN (UniProtKB/Swiss-Prot: P15145.4) was optimised to human codons and synthesised with an N-terminal HA-tag into the plasmid expression vector pTwist CMV BetaGlobin, to create vector pHA-pAPN. Supernatants containing pseudotyped PRCV (ISU1pp) were titrated on HEK293T cells that had been pre-transfected with pHA-APN. After 72 h, firefly luciferase activity was measured using the Bright-Glo system (Promega).

### Neutralization Assays

#### Virus Neutralization

Serum harvested from pigs infected with 135 at 0, 4, 9 and 16 DPI, BAL fluid harvested 16 DPI and supernatant derived from organoid cultures were diluted 1 in 5 in PRCV media, followed by two-fold dilutions up to 1 in 1280. To 100 µl diluted serum, 100 µl of PRCV medium containing 10^3^ PFU of either PRCV 135, 137, ISU-1 or 310, or TEGV 772/70 was added and incubated for 30 min at room temperature with constant agitation. ST cells were inoculated with 100 µl virus/serum mix and incubated for 2 days at 37°C. Cells were fixed with 3.3% formaldehyde and stained with 0.1% crystal violet (H_2_O). The presence of CPE was marked as no neutralization. Each assay was performed in both technical triplicate and in biological triplicate. The neutralization titer that results in 50% neutralization per ml was calculated using the end point calculation method.

#### Pseudo-Virus Neutralization

Serum from PRCV infected pigs and cell culture supernatants from organoids were diluted in cDMEM-10 and 50 µl/well was added to a 96-well plate in triplicate and titrated 2-fold. A dilution of ISU1pp was then added 50 µl/well at a dilution equivalent to between 0.5-1 × 10 ^6^ signal luciferase units and incubated at 37°C for 1 h. Following this, pHA-APN pre-transfected HEK293T target cells were added at a density of 2 x 10 ^4^/100 µL. After 72 h, firefly luciferase activity was measured as described above. Pseudotyped virus neutralisation titers were calculated by interpolating the point at which there was 50% reduction in luciferase activity, relative to untreated controls, neutralisation dose 50% (ND_50_) (33).

### *In Vitro* Organoid Cultures

Fresh cells from TBLN and spleen were cultured as previously described (34). Briefly, cells were resuspended to 6 x 10^7^ cells/ml and plated, 100 µl/well, into Transwell^®^ permeable (0.4 µm pore size) membranes (12 mm diameter polyester membranes in standard 12-well plates Corning), with 1ml complete medium (RPMI with Glutamax, 10% FBS, 1x nonessential amino acids, 1 x sodium pyruvate, 1 x penicillin–streptomycin, 1 x Normocin (*In vivo*Gen) and 1 x insulin/selenium/transferrin cocktail (Gibco)), in the lower chamber. PRCV (MOI 0.01) or medium control was added directly to the insert (cell-containing portion). Cultures were incubated at 37°C in a 5% CO_2_ incubator and supplemented with additional medium to the lower wells if necessary. After 7 days, supernatants were removed for analysis of PRCV-specific antibodies by ELISA, neutralization or pseudo neutralization assays.

### ELISA

96-well Maxisorp ELISA plates (Biolegend, UK) were coated overnight at 4°C with pre-optimized concentrations of ISU-1 full length S protein (1 µg/ml). Two-fold dilutions of serum, either collected pre-inoculation (day 0) or collected post-mortem, starting from 1/20 in PBS-Tween plus Marvel (4%) were applied. Binding of antibodies was detected with goat anti-pig IgG (Fc-specific) conjugated to horseradish peroxidase (AA141P, Biorad, Watford, UK), used at optimal dilution. 1-Step™ Ultra TMB-ELISA Substrate Solution (Thermo Scientific Pierce) was added and the optical density values were read for each well at dual wavelengths (450 nm and 630 nm) using a Labsystems Multiskan plate reader (Fisher Scientific, Loughborough, UK). The quantities of antibody were determined as the reciprocal value of the dilution that gave the first reading above the cut-off value (end-point titer). Cut-off values were determined as mean blank ODs plus 2-fold standard deviations. Starting dilutions were 1:20 for serum, 1:16 for BAL and 1:2 for nasal swab respectively.

### Porcine IFNγ ELISPOT

MultiScreen™-HA ELISPOT plates (Millipore, Watford, UK) were coated overnight at 4°C with 0.5 µg/ml mouse anti-pig IFNγ (BD Biosciences, San Jose, CA) in carbonate buffer (0.1 M Na_2_CO_3_/NaHCO_3_, pH 9.6). Plates were washed 3 times with PBS and subsequently incubated for two hours at 37°C with 200 µl per well of blocking buffer (RPMI, 10% FBS, supplemented with 100 U/ml penicillin and 100 µg/ml streptomycin. The blocking buffer was removed prior to plating stimuli which included medium alone (RPMI-1640 medium with glutamax-I and 25 mM Hepes (Invitrogen Ltd., Paisley, UK) supplemented with penicillin, streptomycin and 10% heat-inactivated FCS, PRCV viruses 135, 137, ISU-1 and 310, peptide pools covering the spike (S) and nucleoprotein (N) of PRCV 135 virus or 2.5 µg/mL Concanavalin A (Con A, Invitrogen). 16 mer peptides overlapping by 12 residues were designed and synthesized by Mimotopes, Melbourne, Australia (**Supplementary Tables 2, 3**). Three pools of peptides representing residues 1-100 (pool 1), 101-200 (pool 2) and 201-305 (pool 3) for the S protein and one pool of 94 for the N protein were used to stimulate T cells at a final concentration of 2 µg/ml.

For each culture condition, triplicate wells of cryopreserved cells (TBLN, BAL, PBMC) at 2 x 10^6^/ml were added to the coated ELISPOT plates (100 µl/well) and cultured for 48 h at 37°C, 5% CO_2_. Cells were washed off the plates using PBS containing 0.05% Tween 20, then 100 µl per well biotinylated mouse anti-pig IFNγ (0.5 µg/ml, BD Biosciences) was added for 2 h at room temperature. Plates were washed five times with PBS containing 0.05% Tween 20 and 100 µl per well streptavidin conjugated to alkaline phosphatase (1/1000, Invitrogen Ltd.) was added for 1 h at room temperature. Plates were washed five times with PBS containing 0.05% Tween 20 and developed with 100 µl per well alkaline phosphatase substrate solution (Biorad laboratories, Hercules, CA) for 20 min at room temperature. Plates were rinsed with tap water and allowed to dry overnight at room temperature before counting the dark blue-coloured immunospots using the AID ELISPOT reader (AID Autoimmun Diagnostika GmbH, Strassberg, Germany). Results were expressed as IFNγ-producing cell number per 10^6^ stimulated cells after subtraction of the average number of spots in media stimulated control wells.

### Intracellular Cytokine Staining

Cryopreserved cells (TBLN, BAL, PBMC) were thawed and seeded at a conc. of 1 x 10^6^ cells/well and stimulated overnight with 135 virus (MOI=0.5) or medium control at 37 °C, 5% CO_2_ before the addition of Brefeldin A (GolgiPlug™, BD Biosciences) allowing accumulation of the cytokine inside the cell. A cocktail of phorbol 12-myristate 13-acetate (PMA)/Ionomycin (Biolegend) was added as a positive control at the same time as the GolgiPlug to separate control wells. Duplicate wells, each containing 1 x 10^6^ cells, were set up for each stimulation condition. After 4 h incubation at 37°C, replicate wells for each stimulation were pooled, centrifuged for 4 min, washed twice with PBS and analyzed for cytokine production using the antibodies listed in **Supplementary Table 4**. Briefly, cells were stained with the primary Abs for surface staining and with Near-Infrared Fixable LIVE/DEAD stain (Invitrogen), for identification of live cells. Following a 20 min incubation at 4°C, cells were washed twice, fixed and permeabilized with BD Cytofix/Cytoperm (BD Biosciences) as per the manufacturer’s instructions. Cells were incubated for 30 min at 4°C with the directly conjugated cytokine antibodies, washed, re-suspended in PBS, and analyzed using a MACSquant analyser10 (Miltenyi). In the case of the IL-2 cytokine antibody, a further incubation step of 20 min at 4°C with the corresponding secondary antibody was necessary since it was not directly conjugated. Thereafter, cells were washed and resuspended in PBS before analysis. Downstream analysis was performed using FlowJo software.

## Results

### Replication of PRCV Strains in Cell Lines

**Figure 1:**
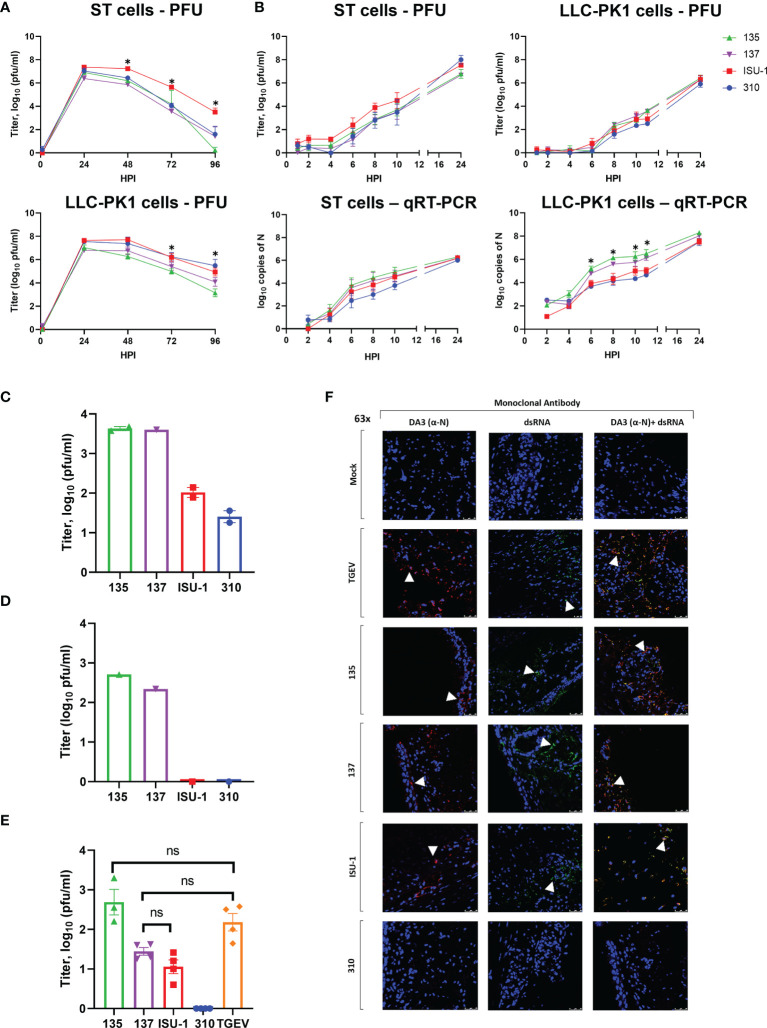
Replication of PRCV strains in cell lines and in *ex vivo* tracheal organ cultures. ST and LLC-PK1 cells were inoculated with 10^4^ PFU (MOI ~ 0.01) of PRCV strains 135, 137, ISU-1 or 310. The quantity of infectious progeny in cell supernatants were determined at 24 h **(A)** or 2 h **(B)** intervals by titration in ST cells. In addition, viral RNA was quantified from cell lysates harvested at the same time points by qRT-PCR using a primers and probes targeting the N gene. In **(A)** and **(B)** each time point represents the mean of three independent experiments with error bars showing the standard error of the mean (SEM). Two-way ANOVA with a Tukey test was used * (p<0.05). For ST cells the star indicates that the ISU-1 titres were higher than those for 137 at 48 - 72 hpi, those for 135 at 48 and 96 hpi, and for 310 at 72 hpi. For LLC-PK1 cells the star indicates that the ISU-1 titres are greater than those for 135 at 72 and 96 hpi. However, **(B)** the quantity of N RNA detected for both 135 and 137 was higher than the RNA detected for to ISU-1 and 310. Tracheal organ cultures (TOCs) were prepared with trachea harvested from 4-week-old piglet in three experiments from separate animals **(C–E)**. TOCs in **(C, D)** were inoculated with 10^4^ PFU of 135, 137, ISU-1 or 310 and the quantity of infectious progeny virus in the supernatants at 24 hpi were determined by titration in ST cells **(C, D)**. **(C)** represents the use of two TOCs and **(D)** a single TOC. **(E)** TOCs were inoculated with 10^5^ PFU of either PRCV 135, 137, 310, ISU-1, TGEV or mock infected with medium only and progeny infectious virus in the supernatants at 24 hpi were determined by titration in ST cells. No virus was detected in the mock control. Statistical differences were evaluated using a one-way ANOVA with a Tukey test for multiple comparisons. No statistical differences (ns) in the amounts of progeny virus produced were identified between ISU-1 & 137, 135 & TGEV or 137 & TGEV; all other comparisons were significant (p<0.05). **(F)** TOCs from **(E)** at 24 hpi were fixed in 4% formalin for 72 h before freezing in OCT. 100 μm cryosections were labelled using anti-N (DA3; Red) and/or anti-dsRNA (Green). Nuclei were stained with DAPI (blue). White arrow indicates the location of detected virus. Images were obtained by a confocal microscope with high powered images (63x magnification) for DA3 only, anti-dsRNA only and co-localization of both DA3 and anti-dsRNA. Scale bars indicate 25μM.

We characterised the *in vitro* replication of four PRCV strains: 135 and 137, both isolated from the respiratory tract of pigs in the UK; 310, isolated in the USA from the small intestine (24) and the previously characterised ISU-1 strain (5, 25). We initially assessed their replication and associated cytopathic effects (CPE) in ST and LLC-PK1 porcine derived cells lines, routinely used for assessment of PRCV and TGEV (**Figures 1A, B****)**. The quantity of infectious progeny was determined by titration in ST cells. Over a 96-h growth period using ST cells, ISU-1 generated higher quantities of infectious progeny than 135, 137 and 310, however statistical significance was only reached in comparison to 137 from 24 – 72 hours post infection (hpi), to 135 from 48 and 96 hpi and to 310 from 72 hpi (p<0.05). In LLC-PK1 cells, 135 generated the lowest titer of virus and was statistically different from ISU-1 from 48 - 96 hpi (p<0.05). Despite these differences, the pattern of replication over 96 h is comparable, with similar peak titers observed at the same time point in both cell types **(** **Figure 1A****).**

No major differences in the quantity of infectious progeny were identified between the four strains in the first 12 h, in either ST or LLC-PK1 cells further indicating that *in vitro* replication was comparable (**Figure 1B**). Infectious progeny present in the cell supernatant was determined by titration. RNA replication in the harvested cells was analysed by quantitative real time PCR (qRT-PCR) using primers and probes targeting the N gene. The quantity of 135 and 137 RNA in LLC-PK1 cells was higher compared to 310 and ISU-1 indicating an increased rate of RNA replication. This however was not observed in ST cells and did not translate into higher numbers of infectious progeny.

### Replication of PRCV 135, 137, ISU-1 and 310 in *Ex Vivo* Tracheal Organ Cultures (TOCs)

Previous work had shown that high titers of PRCV strain TLM83 were isolated from tracheal tissue from experimentally infected pigs, with viral antigen detected in epithelial cells, suggesting active PRCV replication in tracheal epithelial cells (35). To establish whether the four PRCV strains used in this study could replicate in the trachea, primary *ex vivo* TOCs were infected with 1 x 10^4^ PFU of either 135, 137, ISU-1 or 310. The titers of infectious progeny virus from TOCs infected with ISU-1 and 310 were lower than those observed for 135 and 137 in two independent experiments (**Figures 1C, D****)**. Each experiment used TOCs prepared from the trachea harvested from an individual animal and therefore differences may be the result of a host or virus-host interaction effect. In another separate experiment TOCs were inoculated with a log higher titer of 1 x 10^5^ PFU to investigate whether a higher dose was required for the establishment of productive infection for both ISU-1 and 310. Less infectious progeny from ISU-1 and 310 infection was detected in comparison to 135 (p<0.05) (**Figure 1E**). Interestingly the observed titers were comparable between 137 and ISU-1 indicating either ISU-1 requires a higher quantity of input virus to initiate productive replication, or there is a differential host effect, with perhaps trachea extracted from some pigs that are more susceptible to infection than others. TGEV was included in the experiment as previous reports have shown that it is able to replicate in the trachea (36, 37). Titers of TGEV were comparable to those of 135 and 137 strains, confirming the previous observations that TGEV can replicate in the trachea (**Figure 1E**).

To investigate RNA and protein production, the TOCs from the high dose experiment were analysed by confocal microscopy using DA3 and dsRNA antibodies (**Figure 1F**). DA3 was generated from the TGEV nucleoprotein (N) protein and recognizes the four PRCV strains as confirmed by Western blot analysis (28, 38) (**Supplementary Figure 3**). The second antibody targets double stranded RNA (dsRNA) and is a well-established marker for coronavirus RNA replication (32, 39). Both dsRNA and N protein were detected in the mucosal epithelium in TOCs infected with TGEV and PRCV strains 135 and 137 (**Figure 1F**). Small, localized regions of signal for N protein and dsRNA could be detected in TOCs infected with PRCV ISU-1 (**Figure 1F**). No dsRNA or N protein were detected in mock infected TOCs nor in TOCs infected with 310, suggesting that 310 may not be able to replicate in tracheal epithelial cells *in vivo*.

Overall, these data indicated that although the four strains showed comparable replication in ST and LLC-PK1 cells, the 135 and 137 strains replicated more efficiently than ISU-1 and 310 in the tracheal epithelial cells of *ex vivo* TOCs.

### Differences in S Glycoprotein Between PRCV Strains 135, 137, 310 and ISU-1

The replication differences in the *ex vivo* TOCs suggested altered tissue tropism between the four strains. To investigate further, next generation sequencing analysis was performed. As a major determinant of tropism is the S glycoprotein, we compared the amino acid sequence of the four PRCV strains **(** **Supplementary Figure 1**). PRCV 135 and 137 differed by 7 amino acids and 310 and ISU-1 by 9 amino acids. There were a larger number of differences between both 135 and 137 compared to 310- and ISU-1. Interestingly, there is an insertion of amino acids TSV between residue P20 and C21 in 135 and 137, not present in either ISU-1 or 310.

### Analysis of Viral Load After *In Vivo* Inoculation With PRCV Strains 135, 137, 310 and ISU-1

**Figure 2:**
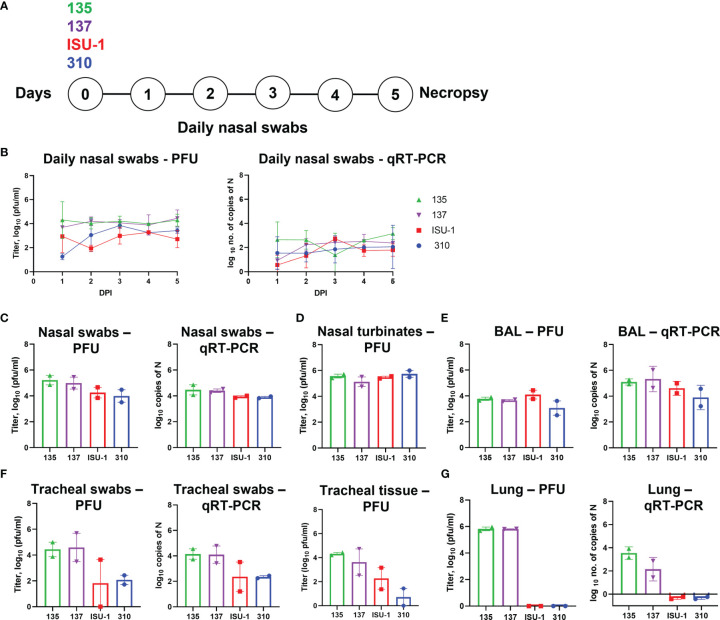
Virus load in tissues after *in vivo* inoculation with PRCV strains 135, 137, 310 and ISU-1. Experimental design **(A)**. Two pigs were infected with 2 x 10^7^ PFU of either 135,137, ISU-1 or 310. All animals were euthanized five days post infection (DPI). Daily nasal swabs were collected **(B)**. At post-mortem nasal swab **(C)**, nasal turbinates **(D)**, BAL **(E)**, tracheal swab and tracheal tissue **(F)** and lung accessory lobe **(G)** were collected. The quantity of infectious virus in the nasal swabs, 0.5 g nasal turbinates, 1 ml BAL fluid, tracheal swab, 0.5 g tracheal tissue and 0.5 g lung accessory lobe was determined by plaque assay. For tracheal tissue, a 3 - 4 mm rings were harvested from the top, middle and bottom of the trachea, and 0.5 g of each ring homogenized; each point represents the mean titers from the three rings.

Following the *ex vivo* analysis we characterized the clinical, virological and pathological parameters of PRCV strains 135, 137, 310 and ISU-1 *in vivo*. Initially a pilot study was carried out using two pigs inoculated with 2 x 10^7^ PFU of each PRCV strain by the intra-nasal/intra-tracheal (IN/IT) route and observed for 5 days (**Figure 2A**). The rectal temperatures of the animals were taken daily and all were within the normal range. At 5 days post-infection (DPI), one PRCV 135 inoculated animal exhibited nasal discharge and coughing, and one PRCV 137 inoculated animal was observed sneezing. Both PRCV 135 animals and the PRCV 137 animal without respiratory signs, displayed signs of lethargy, as did the second animal infected with 137. No clinical signs were observed in PRCV ISU-1 or 310 inoculated animals.

Nasal swabs were taken daily and assessed for presence of infectious virus by titration and PRCV RNA by qRT-PCR of the N gene (**Figure 2B**). The titers of infectious ISU-1 and 310 viruses were lower in the first 2 DPI than 135 and 137 viruses detected in the nasal swabs, suggesting a decrease in productive viral replication. However, the quantities of PRCV derived RNA detected in the nasal swabs were largely similar for all strains. Virus load and the quantity of RNA were comparable between the four strains in nasal swabs, nasal turbinates and BAL fluid taken at post-mortem (5 DPI) (**Figures 2C–E**).

In contrast, the titers of infectious virus and the quantity of PRCV RNA differed between the PRCV strains in the tracheal swab and tracheal tissues taken at post-mortem (5 DPI) (**Figure 2F**). Three segments of trachea, one from the top, middle and bottom of the trachea, were harvested, with no observable difference in virus titer and therefore the mean titer of the three tracheal segments was presented. Less infectious virus was detected in ISU-1 and 310 inoculated animals suggesting that these viruses replicated to a lower level than 135 and 137 in the trachea. No infectious virus for ISU-1 and 310 were detected in the accessory lung lobe, in contrast to 135 and 137 which had titers of ~10^6^ PFU/ml per 0.5 g of tissue (**Figure 2G**). This indicates that no productive replication of 310 and ISU-1 was occurring in the accessory lung lobe, supported by the lack of RNA. No RNA was detected in the blood from any of the four PRCV strains.

These data indicated that ISU-1 and 310 did not replicate efficiently in the trachea and lung of the experimentally inoculated pigs, although they were detected at comparable levels to 135 and 137 strains in nasal swabs, nasal turbinates and BAL at 5 DPI. The patterns of replication for the four PRCV strains *in vivo* in the trachea were comparable to those observed in *ex vivo* TOCs.

### Lung Pathology After *In Vivo* Challenge With PRCV Strains 135, 137, 310 and ISU-1

**Figure 3:**
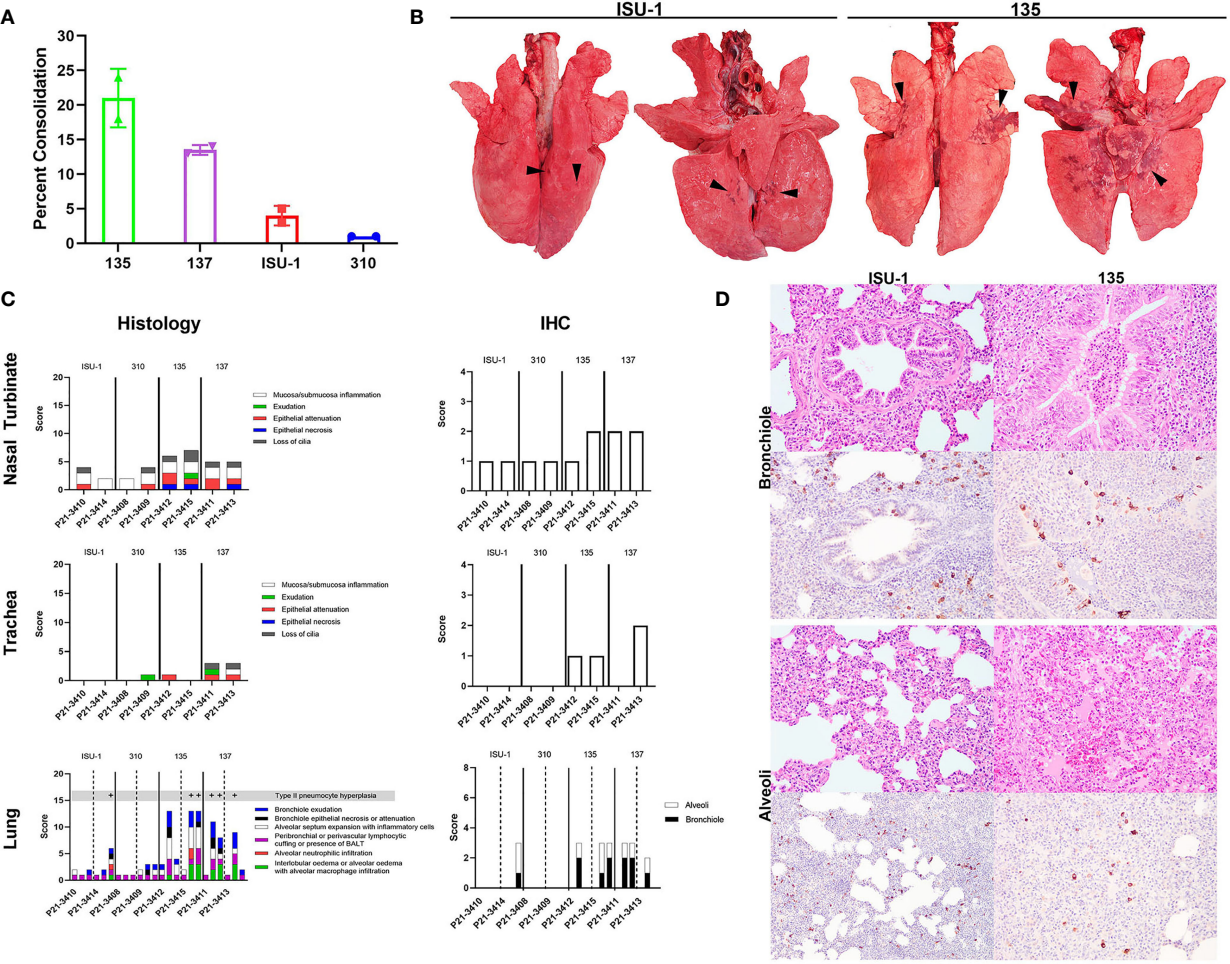
Lung pathology following *in vivo* challenge with PRCV strains 135, 137, ISU-1 and 310. Percent area of lung consolidation **(A)**. Representative gross pulmonary pathology images for pigs infected with either ISU-1 or 135 **(B)**. Arrowheads indicate areas of consolidation. Images are dorsal and ventral views. Histopathology and immunohistochemical scores for nasal turbinate, trachea, and lung **(C)**. Representative histopathology and immunohistochemical (IHC) images of the lung **(D)**. There was only subtle bronchiolar epithelium hyperplasia and association of rare infected bronchiolar epithelium in ISU-1 pig. The bronchiole lumen was filled with neutrophils in 135 infected pig. The alveolar wall of ISU-1 was mildly expanded with inflammatory cells. In the 135 pig, there was multifocal pulmonary oedema and alveolar macrophages, and infrequently intra-alveolar haemorrhage. The N labelled cells within the alveoli were predominantly type II pneumocytes and intra-vascular macrophages, and a small fraction of type I pneumocytes. Images were taken at 200x magnification.

Infected pigs were subjected to necropsy at 5 DPI. On gross examination, infection of the ISU-1 and 310 strains resulted in less than 5% of pulmonary consolidation across all lobes. In contrast, 135 and 137 produced a median of 21% and 13.5%, respectively, of areas of consolidation (**Figure 3A**). The distribution of lesions were characterized by multifocal to coalescing, and were predominantly in middle, caudal, and accessory lung lobes (**Figure 3B**). There were no observable changes within the nasal cavity or the trachea grossly.

The nasal turbinates, trachea and right lung lobes were subjected to histopathology and PRCV N antigen immunohistochemistry (IHC) using DA3 antibody. The changes observed within the nasal turbinates were generally mild, with multifocal lymphoplasmacytic inflammation of the submucosa and occasional intra-epithelial neutrophils (**Figure 3C**). Epithelial necrosis was occasionally observed in pigs infected with 135 or 137, co-localising with N labelling of the mucosa epithelium within areas of lesion. N labelling within the nasal turbinates in pigs infected with ISU-1 or 310 strains were rare. Similarly, in the trachea, microscopic changes were limited to 135 and 137 infected pigs, which exhibited small areas of attenuated epithelium, loss of cilia, which colocalized to areas of virus antigens of the mucosa epithelium. No N labelling was observed in the ISU-1 infected pigs.

The pulmonary changes in ISU-1 and 310 infected pigs were mild, with presence of bronchiole-associated lymphoid tissues (BALT) and occasional expansion of alveolar septum with mononuclear inflammatory cells, representative of antigenic stimulation from viral infection (**Figure 3C**). Only one ISU-1 pig (P21-3414) showed minimal N antigen labelling in bronchiolar epithelium and alveoli.

In the 135 and 137 infected pigs, there was moderate bronchiolar exudation, variably hyperplastic and occasionally attenuated bronchiolar epithelium, and the surrounding airways loosely cuffed by lympho-plasmacytic cells. In the alveoli, there were multifocal to coalescing areas of intra-alveolar oedema, alveolar macrophages, and neutrophils (**Figure 3D**), type II pneumocyte hyperplasia, and the alveolar wall expanded by mononuclear inflammatory cells. Alveolar wall damage was only observed in 135 infected pig (P21-3415). The level of N antigen immunolabelling was moderate within bronchiolar epithelium, and small amounts in type II pneumocytes, intravascular macrophages, alveolar macrophages and occasionally in type I pneumocytes. Overall, the pilot experiment demonstrated that PRCV strains 135 and 137 induced moderate broncho-interstitial pneumonia, while the pathological changes in ISU-1 and 310 were very mild.

### Analysis of Antibody and T Cell Responses After *In Vivo* Inoculation With PRCV Strains 135, 137, 310 and ISU-1

No PRCV specific Ab responses were detected in serum or BAL as measured by ELISA at 5 DPI using recombinant full-length Spike protein or pseudo-neutralization. We enumerated IFNγ secreting cells by ELISpot following re-stimulation with the four PRCV strains or three pools of peptides covering the S protein and one pool of peptides for the N protein of PRCV 135 (**Supplementary Figure 4**, **Supplementary Tables 2, 3**). IFNγ secreting cells were detected by ELISpot in tracheobronchial lymph nodes (TBLN) and peripheral blood mononuclear cells (PBMC). The summed response to the S pool of peptides is shown (**Supplementary Figures 4C, D****)**. The responses to virus stimulation were low whilst there was a greater response to the peptides in TBLN and peripheral blood monocular cells (PBMC) (**Supplementary Figures 4A, B****)**. The responses to the peptides in PBMC were lower compared to TBLN. Overall, it was difficult to establish whether there were differences in the IFNγ responses between the animals infected with different PRCV strains, because too few animals were used, however it was clear that there was a good cross-reactive T cell response at the early time point of 5 DPI.

### Kinetics of Viral Replication After *In Vivo* Inoculation With PRCV 135

**Figure 4:**
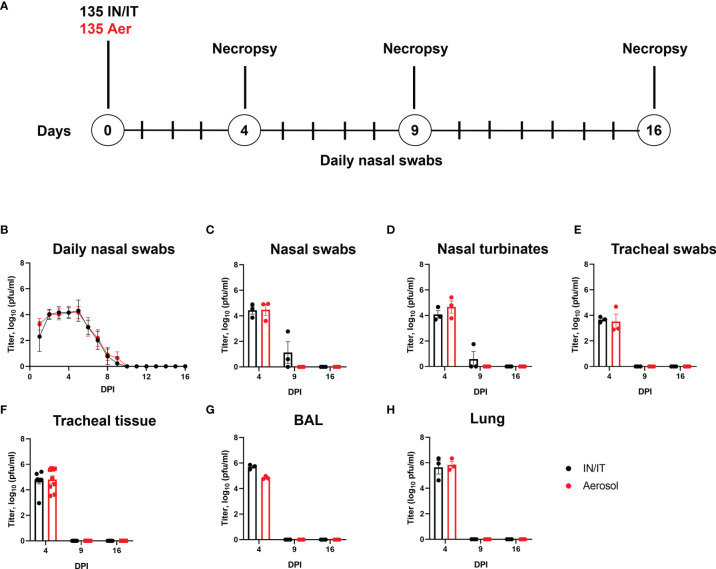
Experimental design and virus load after *in vivo* challenge with PRCV 135. Eighteen pigs were infected with 1 x10^7^ PFU of PRCV 135 by the intratracheal/intranasal route (IN/IT) or by aerosol (Aer). At 4-, 9- and 16-days post infection (DPI) three animals per group were culled **(A)**. Virus load in daily nasal swabs was determined by plaque assays; each point represents the mean titer with error bars indicating SEM **(B)**. At post-mortem nasal swabs **(C),** nasal turbinates **(D)**, tracheal swab **(E)**, tracheal tissue **(F)**, BAL fluid **(G)** and lung accessory lobe **(H)** were harvested, and virus load determined. Each point represents one animal with errors bar defining SEM. Statistical differences at each time point were analysed using a two-way ANOVA with a Tukey test for multiple comparisons; no differences between the groups were identified.

As the observed pathology induced by 135 virus was most severe among the four PRCV strains investigated, we further evaluated the production of infectious virus, pathology and immune responses up to 16 DPI. In addition, we compared two different routes of inoculation – aerosol (Aer) and intra-nasal/intra-tracheal (IN/IT) to determine if this might affect clinical disease, pathology and host responses. Two groups of nine pigs were inoculated with 1 x 10^7^ PFU of PRCV 135 by the IT/IN or by the Aer route using a face mask with Aerogen Solo nebuliser (**Figure 4A****)**. Three animals from each group were culled on 4, 9 and 16 DPI and blood, nasal turbinates, trachea, BAL, mesenteric, retro-pharyngeal and TBLN collected for analysis. Temperatures of above 40°C were recorded in several pigs from both groups as early as 2 DPI, sometimes accompanied with reduced activity, loose faeces and respiratory signs. On only one occasion was a temperature above 41°C recorded (pig 12512, IN/IT group, 3 DPI) and this was accompanied with reduced activity. Six animals from the IN/IT group had loose faeces starting at 4 DPI. Five pigs (one from the IN/IT group and four from the Aer group) showed transient coughing/sneezing between 5 DPI and 8 DPI, in each case resolving within 24 h (**Supplementary Figure 5**). Overall, the clinical signs were mild, detected only in some of the animals, resolved rapidly and never exceeded a score of 5 out of maximum 19 (Clinical signs scoring in **Supplementary Table 1****).**

The presence of infectious 135 virus was assessed by plaque assay from daily nasal swabs. No differences in the amount of virus detected were observed between the IN/IT and Aer groups, with no virus detected after 10 DPI (**Figure 4B**). Analysis of nasal swabs **(** **Figure 4C****),** nasal turbinates **(** **Figure 4D****),** tracheal swab **(** **Figure 4E****),** tracheal tissue **(** **Figure 4F****),** BAL fluid **(** **Figure 4G****)** and lung accessory lobe **(** **Figure 4H****)** also showed there were no differences in the amount of infectious virus detected in the samples between the IN/IT and Aer groups. Although low amounts of virus were detected in the nasal swabs and nasal turbinates in the IN/IT group at 9 DPI no virus was detected in the samples obtained from the Aer group at 9 DPI. The quantity of PRCV derived RNA was not measured in this experiment as the pilot study did not show any difference between the RNA load and infectious viral load determined by titration (**Figure 2**).

Overall the route of inoculation did not affect the amount of infectious virus in any respiratory tissues with no virus detected on or after 9 DPI, although from previous work studying influenza viruses infection of pigs, aerosol delivers only 20-30% of the input dose with uniform distribution throughout the lung compared to IN/IT which delivers a higher proportion to the lung although unevenly, concentrated in a smaller area (40).

### Pathology Following Aerosol and Intra-Nasal/Intra-Tracheal Inoculation With PRCV 135

**Figure 5:**
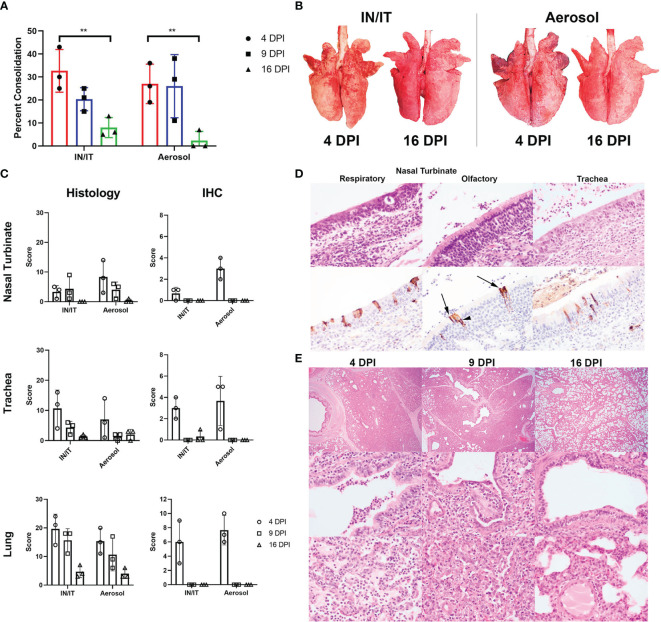
Lung pathology after *in vivo* challenge with PRCV 135. Percent area of lung consolidation **(A)**. Statistical differences are highlighted by ** (p<0.005). Representative gross pulmonary pathology images at 4 and 16 DPI with both routes of infection **(B)**. Histopathology and immunohistochemical (IHC) scores for nasal turbinate, trachea, and lung **(C)**. The scores for each pig are a total of respiratory and olfactory nasal mucosa (nasal turbinate), cranial, middle, and caudal trachea segments (trachea) and right cranial, middle, and caudal lung lobes (lung). Representative histopathology and immunohistochemical images of the respiratory and olfactory component within the nasal turbinate and the trachea from animal P21-3425, aerosol inoculation, 4 DPI **(D)**. Immunolabelling for N in sustentacular (arrow) and olfactory epithelial cells (arrow heads) within the olfactory nasal mucosa. Pulmonary histopathological profile following infection **(E)**. Acute broncho-interstitial pneumonia at 4 DPI with bronchiolar exudation, alveolar oedema and accumulation of alveolar macrophages. The air spaces remained collapsed whereby the alveolar wall is moderately expanded by inflammatory cells at 9 DPI. The bronchiolar epithelium is attenuated but re-epithelisation is also evident. By 16 DPI, the bronchiolar epithelium is hyperplastic, alveolar wall mildly expanded and with loose perivascular cuffing. Sub-gross pulmonary (top row; 20x), bronchiolar (middle row; 400x) and alveolar areas (bottom row; 400x).

Pigs infected with PRCV 135 IN/IT or by Aer were necropsied at 4, 9 and 16 DPI (**Figure 4A**). At 4 DPI animals inoculated by IN/IT or Aer inoculation showed group mean lung consolidation of 32% or 27% respectively, which reduced to 8% and 2% respectively by 16 DPI (**Figures 5A, B**). Histological lesions were mainly detected at 4 and 9 DPI, but N antigen only at 4 DPI (**Figures 5C–E**). The lesions at 4 DPI were predominantly bronchiolar exudation, and within the alveoli were variably oedematous air spaces with neutrophils and alveolar macrophages infiltration. By 9 DPI, there was mild bronchiolar exudation with epithelial attenuation and infrequently necrosis. Occasionally this led to the formation of bronchiolitis obliterans. The alveolar walls were expanded with lymphocytic, plasmacytic and histiocytic cells, and loss of air spaces. By 16 DPI, some residual changes were noted including mild hyperplasia of bronchiole epithelium, and loose perivascular or peri-bronchiolar cuffing with lymphocytes and plasma cells.

The upper respiratory tracts were unremarkable at post-mortem. Histological changes in nasal turbinates were mild, with multifocal inflammation of the submucosa and occasional transmigration of neutrophils across the epithelium in both groups of pigs. However, there was occasional mild epithelial attenuation and exudation in the olfactory nasal mucosa, particularly in the Aer animals at 4 DPI and this frequently colocalized with areas of N labelling. N-labelled cells included olfactory receptors and sustentacular cells. No N immunolabelling was detected at 9 and 16 DPI.

In the trachea, similar lesions and progression comprising mild multifocal lymphoplasmacytic inflammation of the submucosa, occasional epithelial attenuation and exudation were observed (**Figures 5C, D****)** Lesions were more commonly detected and scored higher in the caudal component of the trachea in the Aer group in contrast to the higher scoring in the cranial trachea in the IN/IT group.

Overall, both inoculation methods produced similar moderate pulmonary pathology, with acute lesions and maximal viral infection at 4 DPI and resolution by 16 DPI, similar to known porcine viral pneumonia. In addition, we demonstrated a diversity of cellular tropism within the upper respiratory tract.

### Analysis of Antibody Responses After *In Vivo* Inoculation With PRCV 135

**Figure 6:**
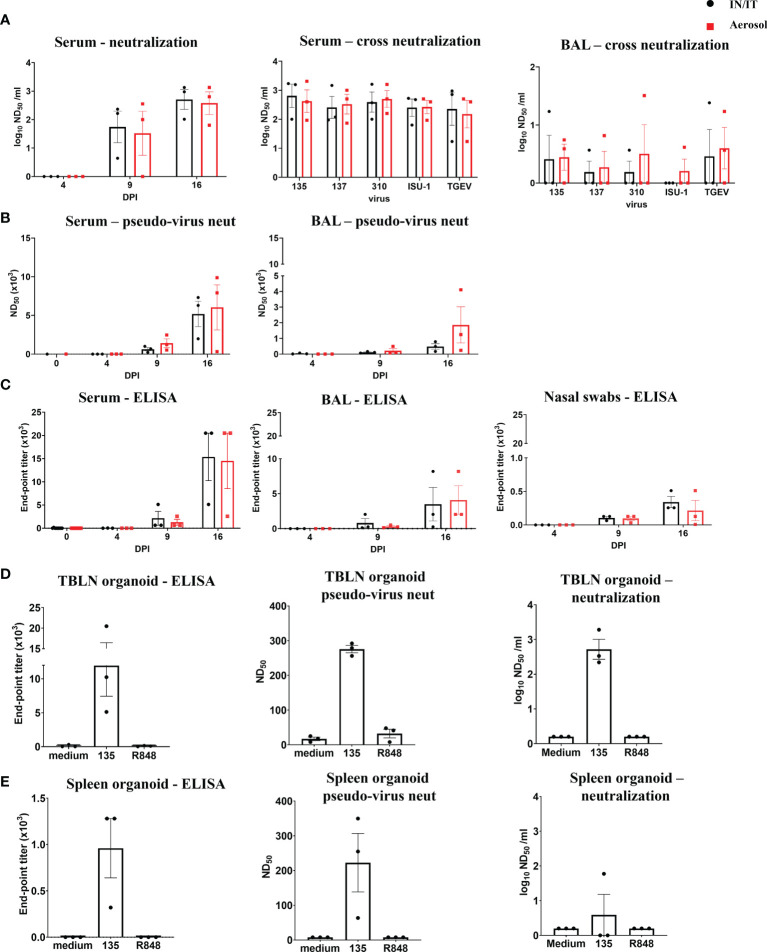
Antibody responses after *in vivo* challenge with PRCV 135. Serum neutralizing titers at 16 DPI were determined by virus neutralization of PRCV135 **(A)**. Serum and BAL were also assayed against 135, 137, ISU-1, 310 and TGEV viruses for cross-neutralization. Pseudo-virus neutralizing dose_50_ titers were determined for serum and BAL at the indicated time points **(B)**. Spike (S) specific IgG responses in serum, BAL and nasal swabs were determined by ELISA at the indicated time points **(C)**. Antibody production after 7 days in organoid culture measured by Spike (S) IgG ELISA, pseudo-neutralization and virus neutralization of PRCV 135 (*n* = 3 pigs from IN/IT from 16 DPI) **(D, E)**. The top of each bar indicates the mean and the line the SEM. Each symbol represents one animal.

The titers of PRCV neutralizing antibodies increased over time in serum and BAL (**Figure 6A****)**. Sera and BAL from both IN/IT and Aer groups cross neutralized against the four PRCV strains and the TGEV strain equally well (**Figure 6A**), indicating that highly cross-reactive antibody responses were generated by both routes of inoculation. A pseudo-virus-based neutralization assay, commonly used for studying SARS-CoV-2 induced responses, was also used to measure PRCV Ab responses and gave similar results to virus neutralization (**Figure 6B**). PRCV specific antibodies were also detected by ELISA using recombinant full-length Spike (S) protein from PRCV ISU-1 strain (**Figure 6C**). S-specific IgG responses were detected at 9 DPI and increased by 16 DPI in serum, broncho-alveolar lavage (BAL) and nasal swabs with no differences between the IN/IT and Aer groups. In addition, we have developed *in vitro* organ cultures with spleen and TBLN harvested at 16 DPI (**Figures 6D, E**) (34). Cultures were stimulated with PRCV 135 or the polyclonal activator R848. After 7 days S-specific IgG was detected in the supernatant in the virus stimulated cultures, which also exhibited both pseudo-virus neutralizing and virus neutralizing activities.

Overall, the data indicate that high titers of cross neutralizing antibodies were produced against the S protein in pigs and *in vitro* systems were developed for the first time in pigs, and used to dissect these responses.

### Analysis of T Cell Responses After *In Vivo* Inoculation With PRCV 135

We enumerated IFNγ secreting cells by ELISpot following stimulation with the four PRCV strains, three pools of peptides covering the S protein and one pool of peptides for the N protein of PRCV 135. Because the IFNγ responses to the stimuli were very similar in the IN/IT and Aer groups, we have shown the grouped responses for all six animals for 4, 9 and 16 DPI. The individual responses by groups are shown in **Supplementary Figures 6, 7**.

**Figure 7:**
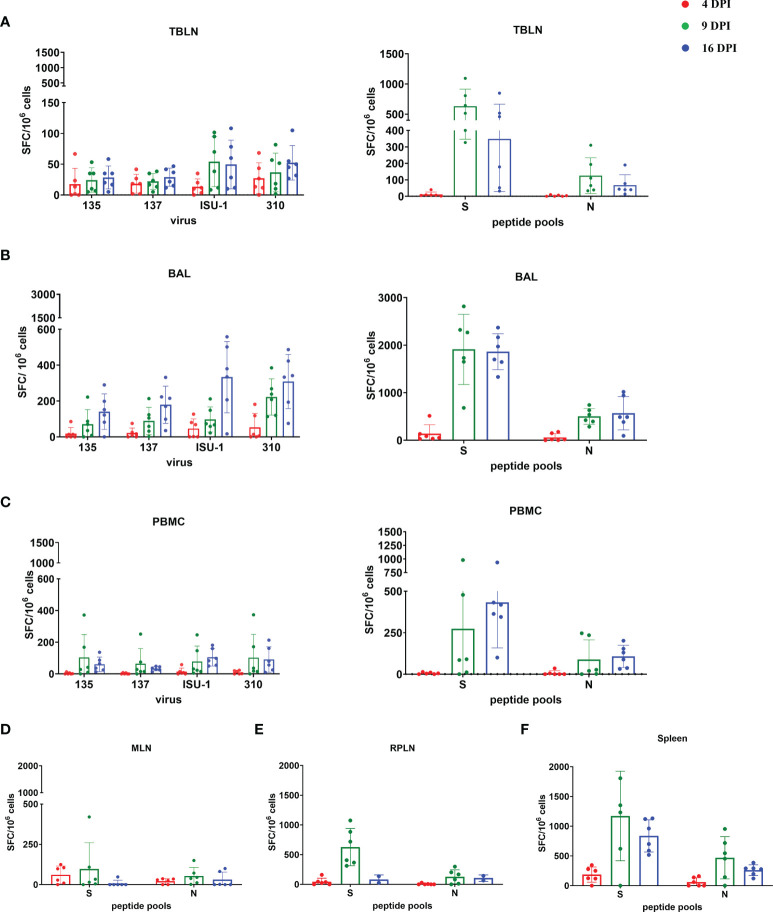
IFNγ ELISpot responses in tissues after *in vivo* challenge with PRCV 135. IFNγ secreting spot forming cells (SFC) were enumerated by ELISpot in tracheobronchial lymph nodes (TBLN) **(A)**, bronchoalveolar lavage (BAL) **(B)**, PBMC **(C)**, mesenteric lymph nodes (MLN) **(D)**, retropharyngeal lymph nodes (RPLN) **(E)** and spleen **(F)** at 4, 9 and 16 DPI, following stimulation with 135, 137, ISU-1 or 310 viruses or pools of peptides covering S and N proteins. Each symbol represents an individual animal, the top of the bar the mean and the line the standard deviation (SD).

The TBLN, BAL and PBMC responses to live virus stimulation were lower compared to the peptide pools, especially to the S protein (**Figures 7A–C**). The summed responses to the three S pools and N peptide pool were highest at 9 and 16 DPI. The responses to the three S peptide pools are shown in **Supplementary Figures 6, 7**. There was animal inter-variability, but it appears that the BAL response, at least to whole virus, was lower reaching a peak at 16 DPI. A similar delayed kinetic of response was observed in BAL of influenza infected animals (30, 41). A response to peptide pools in the spleen, mesenteric and retropharyngeal lymph nodes was detected (**Figures 7D–F**). We also analysed IFNγ, TNF and IL-2 production by CD8β and CD4 T cells in BAL, TBLN and PBMC by intracellular staining (ICS) following stimulation with PRCV 135 virus (**Supplementary Figure 8**). The PRCV 135 specific CD4 and CD8 responses were dominated by IFNγ. The PBMC responses were weak compared to TBLN and BAL.

These data demonstrated that the strongest IFNγ ELISpot response for both virus and S and N peptides was detected in BAL, followed by TBLN and PBMC. There was a good S and N IFNγ ELISpot response in spleen and retropharyngeal lymph nodes, which drain the upper respiratory tract, and a much weaker responses in the distant mesenteric lymph node.

## Discussion

We evaluated the clinical, virological, pathological and immunological parameters of four PRCV strains, 135, 137, 310 and ISU-1. Despite comparable growth characteristics in cell lines, the strains differed in their pathogenicity and replication *in vivo.* ISU-1 and 310 induced mild lung pathology, with reduced replication in the trachea and lungs. In contrast the 135 and 137 strains induced moderate broncho-interstitial pneumonia and replicated efficiently in the trachea and lung. All four strains replicated efficiently in the nasal turbinates. As PRCV 135 infection induced the most severe lung pathology amongst the four strains, we further analysed the time course of infection. Virus was shed until 10 DPI and most pulmonary lesions had resolved by 16 DPI. Cross-reactive Ab and T cell responses were induced in the respiratory tract and systemically.

Currently mouse, ferret, hamster and non-human primate models have been developed to study SARS-CoV-2, but these are not natural host species for infection (19, 20). Mice are not susceptible to Wuhan SARS-CoV-2 isolate because the murine Angiotensin-converting enzyme 2 (ACE-2) does not bind to S glycoprotein. Human ACE2 transgenic mouse cell lines have been developed with different promoters driving ACE2 expression and its tissue distribution and abundance determine the severity of disease (42–44). Ferrets, the gold standard model for influenza virus transmission studies, also transmit SARS-CoV-2 by direct and respiratory contact (45). However, disease severity is mild or absent with viral replication generally restricted to the upper respiratory tract (46). Pathogenesis in non-human primates (NHPs) varies by species. African green monkeys develop acute pneumonia and lung injury persisting for more than a month despite viral clearance, whereas rhesus macaques exhibit pulmonary infiltration like humans with only mild to moderate disease severity (47, 48). However, NHPs are extremely expensive, not easily accessible and raise ethical considerations. Interestingly hamsters can efficiently transmit SARS-CoV2 and experience severe disease including weight loss, diffuse alveolar damage and high viral load in the lung (49, 50). In contrast although the pig cannot be infected with SARS-CoV-2 it is a natural large animal host for several porcine CoVs (5, 51). The pig is physiologically, anatomically and immunologically more similar to humans than small animals and our previous work with influenza showed its utility as a model for human disease (29, 30, 52–55). We also demonstrated that vaccines that protect mice and ferrets against influenza virus are not protective in pigs highlighting differences between small and large animal models (29).

Previously it was suggested that PRCV ISU-1 induced pulmonary pathology resembled that of SARS-CoV (5). However, the lung pathology was mild unless potentiated by immunosuppression with corticosteroid (5, 56). In contrast, we have demonstrated that PRCV strains 135 and 137 induced moderate pulmonary lesions, similar to those of swine influenza infection with transient broncho-interstitial pneumonia and limited alveolar damage (30, 57). These features differ from the pulmonary lesions in severe COVID-19 patients, which typically have diffuse alveolar damage (58). In our experimental pigs, despite moderate severity of PRCV-induced lung lesions, the clinical manifestations of disease were mild and infrequent. Such transient infection, rapid resolution and lung pathology may be similar to humans with mild SARS-CoV-2 infection, while severe disease occurs mainly in elderly with concurrent morbidities.

The cellular tropism of PRCV in the lung encompassed pneumocytes, alveolar macrophages and bronchiolar epithelium, but not vascular tropism such as in severe COVID-19 (59). In the upper respiratory tract, the microscopic exudative rhinitis, observed with PRCV infections in pigs, was similar to other respiratory pathogens such as SARS-CoV-2 in humans and animal models, or swine influenza (60). Examination of the nasal turbinates of PRCV 135 challenged pigs revealed virus replication in the olfactory and sustentacular cells within the olfactory mucosa. There was no further virus invasion into the olfactory nerve fibres nor detection of PRCV N antigen in the olfactory bulb. Olfactory epithelial tropism has been reported for COVID-19 or influenza patients and in experimental animal models (46, 61–64). The olfactory tropism of PRCV in the pig provides a unique opportunity to investigate the pathogenesis of upper respiratory tract infection in a natural host comparable to SARS-CoV-2 infection in humans. Furthermore, the effects of multiple respiratory viral infections such as with PRCV and influenza, or corticosteroid immunosuppression in causing severe acute respiratory disease could be explored (5, 65).

There was no virus replication of 310 or ISU-1 in the accessory lung lobe as determined by titration, although very occasional PRCV N antigen labelling was detected by immunohistochemistry. This inefficient virus replication may account for the differences in lung pathology. PRCV uses APN as a receptor (11, 66). Although the distribution of APN in the pig tissue has not been characterised, a recent study using primary porcine tracheal and bronchial epithelial cells and trachea tissue demonstrated low levels of APN expression (67). However, the *in vitro* study by Peng et al, also demonstrated the greater susceptibility of bronchial cells compared to tracheal cells was not APN dependant, suggesting another host entry or host restriction factor. The entry of CoVs is mediated by the S glycoprotein, a heavily glycosylated type I transmembrane, class I fusion protein (68). The change of tropism between TGEV which infects the gastroenteric tract and PRCV which infects the respiratory tract has been attributed to a 200 amino acid deletion in the S1 region of the S glycoprotein which removes the ability to bind sialic acid (9, 10, 67). Changes in tropism for other CoV including Mouse Hepatitis Virus, Infectious Bronchitis Virus and SARS-CoV have all been attributed to changes in the S (69–72). The S glycoproteins of PRCV strains 135 and 137 are three amino acids longer when compared to the 310 and ISU-1 (**Supplementary Figure 1**). There is an insertion of the amino acids TSV between residue P20 and P21 of the ISU-1 reference sequence (accession number DQ811787) in 135 and 137. Additionally, there are other amino acid differences in the S protein located in the S1 region that mediates receptor binding. It is therefore possible, that the differences in the PRCV S proteins are responsible for the differences in observed tissue tropism, which might be APN independent. The use of TOC cultures, which mimicked the replication pattern *in vivo* may help to dissect the mechanisms of PRCV tissue tropism.

It is also possible that the differences in *in vivo* replication and pathology are not virus entry related but related to gene 3. The size of the deletion in gene 3 has been hypothesized to relate to the varying pathogenicity of different PRCV strains, however the role of gene 3 in virulence is not clear (31, 73). Although a small plaque mutant of the virulent TGEV Miller strain, with a large deletion in gene 3 was attenuated, deleting gene 3 from the TGEV Purdue strain had little effect on virulence with the recombinant virus retaining replication efficiency and tropism of wild type virus (74, 75). Sequence analysis of gene 3 of the four PRCV strains demonstrated varying sized deletions in both open reading frame (ORF) 3a and 3b (**Supplementary Figure 2****).** Notably the deletion in all four strains has removed the reported canonical transcription regulatory sequence (TRS) of ACTAAAC (76) and the later published core TRS sequence of CTAAAC, although a partial sequence of AAC remains (77). According to the model of discontinuous transcription for the production of the structural and accessory genes proposed by Sawicki and Sawicki, this is likely not sufficient for subgenomic mRNA (sgmRNA) synthesis however a non-canonical TRS of CAA has been reported for gene 4b in IBV (78, 79). The ATG start codon for 3a however is deleted in all four strains. ORF 3b is interesting as both 135 and 137 have a TRS of ACTAAAC upstream of the 3b start codon, first reported by Page et al, which is a result of two mutations. ISU-1, 310 and TGEV have a sequence of TCTAAAT. There is an ATG start codon downstream of the TRS which could hypothetically produce a protein in both 135 and 137 of 244 amino acids, the same as 3b in TGEV. There are seven amino acids difference in comparison to the TGEV Purdue reference sequence (accession number DQ811788). Interestingly the amino acid sequence of 3b in the isolate of ISU-1 used in this study is different to that of the reference sequence, and therefore potentially different to that used by Jung et al., 2007. If a sgmRNA is produced from the sequence TCTAAAT, the 3b protein is truncated due to a premature stop codon however there is a second ATG which does hypothetically produce a second protein which aligns with the 5’ end of the 3b protein. Further investigation is required to determine whether the 3b protein is produced and whether it is involved in causing the different pathogenicity of the four strains.

Infected animals produced cross neutralizing antibody, in both serum and BAL, against the four PRCV strains and TGEV. In addition, we established for the first-time porcine spleen and pulmonary lymph node organoid cultures which reproduced the Ab response as observed *in vivo*. However further studies will establish their utility to dissect the Ab and B cell responses. Induction of effective early immune control and durable immune memory is critical for protection against severe disease and reinfection with CoVs. Some studies have suggested that there is an impaired germinal centre reaction in acute Covid 19 infection and others that neutralizing Ab titers wane rapidly after mild infection (80–82). We have only measured responses until 16 DPI, further studies will be required to establish the duration and protective efficacy of the PRCV Ab responses *in vivo*. Infection of adult swine with TGEV induced serum antibodies persisting for six months to several years (51). PRCV-inoculated pigs have been reported to develop potent systemic and bronchus-associated but not gut associated antibody and antibody secreting cell (ASC) responses (83, 84). Despite the lack of gut associated antibody, the decline of TGEV has been widely attributed to cross-immunity following PRCV infection (7, 85, 86). Relocation of PRCV IgG and IgA ASC from the BALT to the gut of the PRCV-exposed pigs after TGEV challenge has been suggested to account for the induced partial protection (87).

In our experiments the strongest T cell response was found in BAL, as we have previously shown for pigs infected with influenza virus (30, 41). IFNγ production was detected by IFNγ ELISpot following stimulation with S and N peptides. The ability to dissect the Ab, B and T cell responses in the respiratory tract in pigs, which is limited in humans is a real advantage of the swine model. Although we have not shown that PRCV-specific T cells are protective, human data suggests that T cells specific for the replication/transcription complex are associated with protection against SARS-CoV-2 infection in repeatedly exposed healthcare workers (88). Similarly, a T cell response to an immunodominant N_105-113_ epitope in B*07:02 individuals correlates with less severe disease (89). The PRCV ISU-1 pathology in pigs induced by corticosteroid treatment was also associated with reduced CD4 and CD8 T cell numbers in pulmonary lesions and ineffective virus clearance (5). In contrast to the beneficial effects of T cells producing effector cytokines, early production of less pro-inflammatory cytokines in BAL correlated with the reduced pathogenicity of PRCV, compared to swine influenza or porcine reproductive respiratory syndrome virus (6). Further studies to delineate more fully the frequency, specificity, functionality, and durability of T responses in pigs infected with pathogenic and apathogenic PRCVs, may reveal critical features of the T cell response.

We have described virological, pathological and immunological characteristics of PRCV strains with differing pathogenicity. Based on our experience with the pig as a model for human influenza we suggest that detailed analysis of the pathogenesis and immune control of infection with these PRCV strains will provide important insights into the causes of mild or severe coronavirus-induced respiratory diseases. These insights will be crucial for the development of better containment strategies for emerging porcine coronavirus diseases, prevention of zoonotic transmission to humans and prevention and treatment of human coronavirus diseases.

## Data Availability Statement

The datasets presented in this study can be found in online repositories. The names of the repository/repositories and accession number(s) can be found below: https://www.ncbi.nlm.nih.gov/genbank/, accession numbers OM830318, OM830319, OM830320 and OM830321.

## Ethics Statement

The animal study was reviewed and approved by Animal Welfare Ethical Review Body at the Pirbright Institute and Animal and Plant Health Agency. The study was conducted according to the U.K. Government Animal (Scientific Procedures) Act 1986 under project license PP7764821.

## Author Contributions

Conceptualization: ET, EB, BC, TT, JH, and RW. Methodology: SK, BVC, AF, JN, GD, IW, EV, AM, BP, NT, DB, ET, EB, HM, RM, FL, PB, and HE. Investigation: ET, EB, BVC, and SK. Sequencing: GF and NP. Pathology: FL and AN. Funding acquisition: ET, BC, JH, EB, and TT. Supervision: ET, EB, and SK. Writing – original draft: ET, SK, FL, and BVC. Writing – review and editing: PB, AN, BC, NT, and GF. All authors contributed to the article and approved the submitted version.

## Funding

This work was supported by the UKRI Biotechnology and Biological Sciences Research Council (BBSRC) BBS/E/I/00007030, BBS/E/00007031, and BBS/E/I/00007038 BBS/E/I/00007039.

## Conflict of Interest

RM is an employee of company Aerogen.

The remaining authors declare that the research was conducted in the absence of any commercial or financial relationships that could be construed as a potential conflict of interest.

## Publisher’s Note

All claims expressed in this article are solely those of the authors and do not necessarily represent those of their affiliated organizations, or those of the publisher, the editors and the reviewers. Any product that may be evaluated in this article, or claim that may be made by its manufacturer, is not guaranteed or endorsed by the publisher.

## References

[1] Kenney SP Wang Q Vlasova A Jung K Saif L. Naturally Occurring Animal Coronaviruses as Models for Studying Highly Pathogenic Human Coronaviral Disease. Vet Pathol (2021) 58(3):438–52. doi: 10.1177/0300985820980842 33357102

[2] Lednicky JA Tagliamonte MS White SK Elbadry MA Alam MM Stephenson CJ. Emergence of Porcine Delta-Coronavirus Pathogenic Infections Among Children in Haiti Through Independent Zoonoses and Convergent Evolution. medRxiv (2021) 2021.03.19.21253391. doi: 10.1101/2021.03.19.21253391

[3] Pensaert M Callebaut P Vergote J. Isolation of a Porcine Respiratory, non-Enteric Coronavirus Related to Transmissible Gastroenteritis. Vet Q (1986) 8(3):257–61. doi: 10.1080/01652176.1986.9694050 3018993

[4] Doyle LP Hutchings LM. A Transmissible Gastroenteritis in Pigs. J Am Vet Med Assoc (1946) 108:257–9. 21020443

[5] Jung K Alekseev KP Zhang X Cheon DS Vlasova AN Saif LJ. Altered Pathogenesis of Porcine Respiratory Coronavirus in Pigs Due to Immunosuppressive Effects of Dexamethasone: Implications for Corticosteroid Use in Treatment of Severe Acute Respiratory Syndrome Coronavirus. J Virol (2007) 81(24):13681–93. doi: 10.1128/JVI.01702-07 PMC2168842 17942563

[6] Van Reeth K Labarque G Nauwynck H Pensaert M. Differential Production of Proinflammatory Cytokines in the Pig Lung During Different Respiratory Virus Infections: Correlations With Pathogenicity. Res Vet Sci (1999) 67(1):47–52. doi: 10.1053/rvsc.1998.0277 10425240 PMC7126504

[7] Saif LJ van Cott JL Brim TA. Immunity to Transmissible Gastroenteritis Virus and Porcine Respiratory Coronavirus Infections in Swine. Vet Immunol Immunopathol (1994) 43(1-3):89–97. doi: 10.1016/0165-2427(94)90124-4 7856068 PMC7119820

[8] Rasschaert D Duarte M Laude H. Porcine Respiratory Coronavirus Differs From Transmissible Gastroenteritis Virus by a Few Genomic Deletions. J Gen Virol (1990) 71(Pt 11):2599–607. doi: 10.1099/0022-1317-71-11-2599 2174956

[9] Schultze B Krempl C Ballesteros ML Shaw L Schauer R Enjuanes L. Transmissible Gastroenteritis Coronavirus, But Not the Related Porcine Respiratory Coronavirus, has a Sialic Acid (N-Glycolylneuraminic Acid) Binding Activity. J Virol (1996) 70(8):5634–7. doi: 10.1128/jvi.70.8.5634-5637.1996 PMC190524 8764078

[10] Krempl C Schultze B Laude H Herrler G. Point Mutations in the s Protein Connect the Sialic Acid Binding Activity With the Enteropathogenicity of Transmissible Gastroenteritis Coronavirus. J Virol (1997) 71(4):3285–7. doi: 10.1128/jvi.71.4.3285-3287.1997 PMC191465 9060696

[11] Delmas B Gelfi J Kut E Sjöström H Noren O Laude H. Determinants Essential for the Transmissible Gastroenteritis Virus-Receptor Interaction Reside Within a Domain of Aminopeptidase-N That is Distinct From the Enzymatic Site. J Virol (1994) 68(8):5216–24. doi: 10.1128/jvi.68.8.5216-5224.1994 PMC236465 7913510

[12] Delmas B Gelfi J Sjöström H Noren O Laude H. Further Characterization of Aminopeptidase-N as a Receptor for Coronaviruses. In: Laude H Vautherot J-F, editors. Coronaviruses: Molecular Biology and Virus-Host Interactions. Boston, MA: Springer US (1993). p. 293–8. 10.1007/978-1-4615-2996-5_45 7911642

[13] Page KW Mawditt KL Britton P. Sequence Comparison of the 5’ End of Mrna 3 From Transmissible Gastroenteritis Virus and Porcine Respiratory Coronavirus. J Gen Virol (1991) 72(Pt 3):579–87. doi: 10.1099/0022-1317-72-3-579 1848593

[14] Laude H Van Reeth K Pensaert M. Porcine Respiratory Coronavirus: Molecular Features and Virus-Host Interactions. Vet Res (1993) 24(2):125–50. 8393722

[15] Wesley RD Woods RD Cheung AK. Genetic Analysis of Porcine Respiratory Coronavirus, an Attenuated Variant of Transmissible Gastroenteritis Virus. J Virol (1991) 65(6):3369–73. doi: 10.1128/jvi.65.6.3369-3373.1991 PMC240999 1851885

[16] Chen F Knutson TP Rossow S Saif LJ Marthaler DG. Decline of Transmissible Gastroenteritis Virus and its Complex Evolutionary Relationship With Porcine Respiratory Coronavirus in the United States. Sci Rep (2019) 9(1):3953. doi: 10.1038/s41598-019-40564-z 30850666 PMC6408454

[17] Saif LJ. Animal Coronaviruses: What can They Teach Us About the Severe Acute Respiratory Syndrome? Rev Sci Tech (2004) 23(2):643–60. doi: 10.20506/rst.23.2.1513 15702725

[18] Halbur PG Paul PS Vaughn EM Andrews JJ. Experimental Reproduction of Pneumonia in Gnotobiotic Pigs With Porcine Respiratory Coronavirus Isolate AR310. J Vet Diagn Invest (1993) 5(2):184–8. doi: 10.1177/104063879300500207 8389599

[19] Muñoz-Fontela C Dowling WE Funnell SGP Gsell PS Riveros-Balta AX Albrecht RA. Animal Models for COVID-19. Nature (2020) 586(7830):509–15. doi: 10.1038/s41586-020-2787-6 PMC8136862 32967005

[20] Flerlage T Boyd DF Meliopoulos V Thomas PG Schultz-Cherry S. Influenza Virus and SARS-Cov-2: Pathogenesis and Host Responses in the Respiratory Tract. Nat Rev Microbiol (2021) 19(7):425–41. doi: 10.1038/s41579-021-00542-7 PMC8023351 33824495

[21] Rajao DS Vincent AL. Swine as a Model for Influenza a Virus Infection and Immunity. ILAR J (2015) 56(1):44–52. doi: 10.1093/ilar/ilv002 25991697

[22] Judge EP Hughes JM Egan JJ Maguire M Molloy EL O’Dea S. Anatomy and Bronchoscopy of the Porcine Lung. A Model for Translational Respiratory Medicine. Am J Respir Cell Mol Biol (2014) 51(3):334–43. doi: 10.1165/rcmb.2013-0453TR 24828366

[23] Pabst R. The Pig as a Model for Immunology Research. Cell Tissue Res (2020) 380(2):287–304. doi: 10.1007/s00441-020-03206-9 32356014 PMC7223737

[24] Vaughn EM Halbur PG Paul PS. Three New Isolates of Porcine Respiratory Coronavirus With Various Pathogenicities and Spike (s) Gene Deletions. J Clin Microbiol (1994) 32(7):1809–12. doi: 10.1128/jcm.32.7.1809-1812.1994 PMC263803 7929779

[25] Hill HT Biwer JD Wood RD Wesley RD. Porcine Respiratory Coronavirus Isolated From Two U.s. Swine Herds. In: Neuzil TA, editor. Proceedings of the American Association of Swine Practitioners. Des Moines, IA: American Association of Swine Practitioners (1989). p. 333–5.

[26] Brown I Cartwright S. New Porcine Coronavirus? Vet Rec (1986) 119(11):282–3. doi: 10.1136/vr.119.11.282 3022457

[27] Garwes DJ Pocock DH. The Polypeptide Structure of Transmissible Gastroenteritis Virus. J Gen Virol (1975) 29(1):25–34. doi: 10.1099/0022-1317-29-1-25 171335

[28] Martín Alonso JM Balbín M Garwes DJ Enjuanes L Gascón S Parra F. Antigenic Structure of Transmissible Gastroenteritis Virus Nucleoprotein. Virology (1992) 188(1):168–74. doi: 10.1016/0042-6822(92)90746-C PMC7130495 1373552

[29] Holzer B Morgan SB Matsuoka Y Edmans M Salguero FJ Everett H. Comparison of Heterosubtypic Protection in Ferrets and Pigs Induced by a Single-Cycle Influenza Vaccine. J Immunol (2018) 200(12):4068–77. doi: 10.4049/jimmunol.1800142 PMC5985365 29703861

[30] Martini V Paudyal B Chrun T McNee A Edmans M Atangana Maze E. Simultaneous Aerosol and Intramuscular Immunization With Influenza Vaccine Induces Powerful Protective Local T Cell and Systemic Antibody Immune Responses in Pigs. J Immunol (2021) 206(3):652–63. doi: 10.4049/jimmunol.2001086 PMC7812058 33328212

[31] Halbur PG Paul PS Frey ML Landgraf J Eernisse K Meng XJ. Comparison of the Pathogenicity of Two US Porcine Reproductive and Respiratory Syndrome Virus Isolates With That of the Lelystad Virus. Vet Pathol (1995) 32(6):648–60. doi: 10.1177/030098589503200606 8592800

[32] Lean FZX Lamers MM Smith SP Shipley R Schipper D Temperton N. Development of Immunohistochemistry and *in Situ* Hybridisation for the Detection of SARS-Cov and SARS-Cov-2 in Formalin-Fixed Paraffin-Embedded Specimens. Sci Rep (2020) 10(1):21894. doi: 10.1038/s41598-020-78949-0 33318594 PMC7736337

[33] Thakur N Gallo G Elreafey AME Bailey D. Production of Recombinant Replication-Defective Lentiviruses Bearing the SARS-Cov or SARS-Cov-2 Attachment Spike Glycoprotein and Their Application in Receptor Tropism and Neutralisation Assays. Bio Protoc (2021) 11(21):e4249. doi: 10.21769/BioProtoc.4249 PMC8595443 34859135

[34] Wagar LE Salahudeen A Constantz CM Wendel BS Lyons MM Mallajosyula V. Modeling Human Adaptive Immune Responses With Tonsil Organoids. Nat Med (2021) 27(1):125–35. doi: 10.1038/s41591-020-01145-0 PMC7891554 33432170

[35] Cox E Hooyberghs J Pensaert MB. Sites of Replication of a Porcine Respiratory Coronavirus Related to Transmissible Gastroenteritis Virus. Res Vet Sci (1990) 48(2):165–9. doi: 10.1016/S0034-5288(18)30984-6 PMC7130871 2159175

[36] Frederick GT Bohl EH. Local and Systemic Cell-Mediated Immunity Against Transmissible Gastroenteritis, an Intestinal Viral Infection of Swine. J Immunol (1976) 116(4):1000–4. 768378

[37] Furuchi S Shimizu Y Kymagap T. Multiplication of Low and High Cell Culture Passaged Strains of Transmissible Gastroenteritis Virus in Organs of Newborn Piglets*. Vet Microbiol (1978) 3:169–78. doi: 10.1016/0378-1135(79)90033-6

[38] Garwes DJ Stewart F Elleman CJ. Identification of Epitopes of Immunological Importance on the Peplomer of Por- Cine Transmissible Gastroenteritis Virus. In: Lai M Stohlman S, editors. Coronaviruses. Plenum, New York: Plenum Press (1987). p. 509–16. 10.1007/978-1-4684-1280-2_66 2449047

[39] Doyle N Simpson J Hawes PC Maier HJ. Coronavirus RNA Synthesis Takes Place Within Membrane-Bound Sites. Viruses (2021) 13(12):2540. doi: 10.3390/v13122540 34960809 PMC8708976

[40] Martini V Hinchcliffe M Blackshaw E Joyce M McNee A Beverley P. Distribution of Droplets and Immune Responses After Aerosol and Intra-Nasal Delivery of Influenza Virus to the Respiratory Tract of Pigs. Front Immunol (2020) 11:594470. doi: 10.3389/fimmu.2020.594470 33193445 PMC7653178

[41] Martini V Edmans M Gubbins S Jayaraman S Paudyal B Morgan S. Spatial, Temporal and Molecular Dynamics of Swine Influenza Virus-Specific CD8 Tissue Resident Memory T Cells. Mucosal Immunol (2022). doi: 10.1038/s41385-021-00478-4 PMC9038527 35145208

[42] Gu H Chen Q Yang G He L Fan H Deng YQ. Adaptation of SARS-Cov-2 in BALB/C Mice for Testing Vaccine Efficacy. Science (2020) 369(6511):1603–7. doi: 10.1126/science.abc4730 PMC7574913 32732280

[43] Leist SR Dinnon KH 3rd Schäfer A Tse LV Okuda K Hou YJ. A Mouse-Adapted SARS-Cov-2 Induces Acute Lung Injury and Mortality in Standard Laboratory Mice. Cell (2020) 183(4):1070–85.e12. doi: 10.1016/j.cell.2020.09.050 33031744 PMC7510428

[44] Oladunni FS Park JG Pino PA Gonzalez O Akhter A Allué-Guardia A. Lethality of SARS-Cov-2 Infection in K18 Human Angiotensin-Converting Enzyme 2 Transgenic Mice. Nat Commun (2020) 11(1):6122. doi: 10.1038/s41467-020-19891-7 33257679 PMC7705712

[45] Richard M Kok A de Meulder D Bestebroer TM Lamers MM Okba NMA. SARS-Cov-2 is Transmitted *via* Contact and *via* the Air Between Ferrets. Nat Commun (2020) 11(1):3496. doi: 10.1038/s41467-020-17367-2 32641684 PMC7343828

[46] Everett HE Lean FZX Byrne AMP van Diemen PM Rhodes S James J. Intranasal Infection of Ferrets With SARS-Cov-2 as a Model for Asymptomatic Human Infection. Viruses (2021) 13(1). doi: 10.3390/v13010113 PMC7830262 33467732

[47] Munster VJ Feldmann F Williamson BN van Doremalen N Pérez-Pérez L Schulz J. Respiratory Disease in Rhesus Macaques Inoculated With SARS-Cov-2. Nature (2020) 585(7824):268–72. doi: 10.1038/s41586-020-2324-7 PMC7486227 32396922

[48] Hild SA Chang MC Murphy SJ Grieder FB. Nonhuman Primate Models for SARS-Cov-2 Research: Infrastructure Needs for Pandemic Preparedness. Lab Anim (2021) 50(6):140–1. doi: 10.1038/s41684-021-00760-9 33927412

[49] Sia SF Yan L-M Chin AWH Fung K Choy K-T Wong AYL. Pathogenesis and Transmission of SARS-Cov-2 in Golden Hamsters. Nature (2020) 583(7818):834–8. doi: 10.1038/s41586-020-2342-5 PMC7394720 32408338

[50] Chan JF Zhang AJ Yuan S Poon VK Chan CC Lee AC. Simulation of the Clinical and Pathological Manifestations of Coronavirus Disease 2019 (COVID-19) in a Golden Syrian Hamster Model: Implications for Disease Pathogenesis and Transmissibility. Clin Infect Dis (2020) 71(9):2428–46. doi: 10.1093/cid/ciaa325 PMC7184405 32215622

[51] Vlasova AN Wang Q Jung K Langel SN Malik YS Saif LJ. Porcine Coronaviruses. Emerging Transboundary Anim Viruses (2020) 79–110. doi: 10.1007/978-981-15-0402-0_4

[52] Holzer B Morgan SB Martini V Sharma R Clark B Chiu C. Immunogenicity and Protective Efficacy of Seasonal Human Live Attenuated Cold-Adapted Influenza Virus Vaccine in Pigs. Front Immunol (2019) 10:2625. doi: 10.3389/fimmu.2019.02625 31787986 PMC6856147

[53] McNee A Smith TRF Holzer B Clark B Bessell E Guibinga G. Establishment of a Pig Influenza Challenge Model for Evaluation of Monoclonal Antibody Delivery Platforms. J Immunol (2020) 205(3):648–60. doi: 10.1101/2020.03.12.988808 PMC7372317 32591390

[54] Edmans M McNee A Porter E Vatzia E Paudyal B Martini V. Magnitude and Kinetics of T Cell and Antibody Responses During H1n1pdm09 Infection in Inbred Babraham Pigs and Outbred Pigs. Front Immunol (2021) 11(3708). doi: 10.3389/fimmu.2020.604913 PMC7884753 33603740

[55] Canini L Holzer B Morgan S Dinie Hemmink J Clark B Woolhouse MEJ. Timelines of Infection and Transmission Dynamics of H1n1pdm09 in Swine. PloS Pathog (2020) 16(7):e1008628. doi: 10.1371/journal.ppat.1008628 32706830 PMC7446876

[56] Jung K Renukaradhya GJ Alekseev KP Fang Y Tang Y Saif LJ. Porcine Reproductive and Respiratory Syndrome Virus Modifies Innate Immunity and Alters Disease Outcome in Pigs Subsequently Infected With Porcine Respiratory Coronavirus: Implications for Respiratory Viral Co-Infections. J Gen virol (2009) 90(Pt 11):2713–23. doi: 10.1099/vir.0.014001-0 PMC2862479 19656969

[57] Janke BH. Influenza a Virus Infections in Swine: Pathogenesis and Diagnosis. Vet Pathol (2013) 51(2):410–26. doi: 10.1177/0300985813513043 24363301

[58] Caramaschi S Kapp ME Miller SE Eisenberg R Johnson J Epperly G. Histopathological Findings and Clinicopathologic Correlation in COVID-19: A Systematic Review. Modern Pathol (2021) 34(9):1614–33. doi: 10.1038/s41379-021-00814-w PMC8141548 34031537

[59] Schurink B Roos E Radonic T Barbe E Bouman CSC de Boer HH. Viral Presence and Immunopathology in Patients With Lethal COVID-19: A Prospective Autopsy Cohort Study. Lancet Microbe (2020) 1(7):e290–e9. doi: 10.1016/S2666-5247(20)30144-0 PMC7518879 33015653

[60] De Vleeschauwer A Atanasova K Van Borm S van den Berg T Rasmussen TB Uttenthal Å. Comparative Pathogenesis of an Avian H5N2 and a Swine H1N1 Influenza Virus in Pigs. PloS One (2009) 4(8):e6662. doi: 10.1371/journal.pone.0006662 19684857 PMC2722722

[61] Meinhardt J Radke J Dittmayer C Franz J Thomas C Mothes R. Olfactory Transmucosal SARS-Cov-2 Invasion as a Port of Central Nervous System Entry in Individuals With COVID-19. Nat Neurosci (2021) 24(2):168–75. doi: 10.1101/2020.06.04.135012 33257876

[62] Schrauwen EJA Herfst S Leijten LM Run Pv Bestebroer TM Linster M. The Multibasic Cleavage Site in H5N1 Virus is Critical for Systemic Spread Along the Olfactory and Hematogenous Routes in Ferrets. J Virol (2012) 86(7):3975–84. doi: 10.1128/JVI.06828-11 PMC3302532 22278228

[63] Khan M Yoo SJ Clijsters M Backaert W Vanstapel A Speleman K. Visualizing in Deceased COVID-19 Patients How SARS-Cov-2 Attacks the Respiratory and Olfactory Mucosae But Spares the Olfactory Bulb. Cell (2021) 184(24):5932–49.e15. doi: 10.1016/j.cell.2021.10.027 34798069 PMC8564600

[64] van Riel D Verdijk R Kuiken T. The Olfactory Nerve: A Shortcut for Influenza and Other Viral Diseases Into the Central Nervous System. J Pathol (2015) 235(2):277–87. doi: 10.1002/path.4461 25294743

[65] Bai L Zhao Y Dong J Liang S Guo M Liu X. Coinfection With Influenza a Virus Enhances SARS-Cov-2 Infectivity. Cell Res (2021) 31(4):395–403. doi: 10.1038/s41422-021-00473-1 33603116 PMC7890106

[66] Delmas B Gelfi J L’Haridon R Vogel LK Sjöström H Norén O. Aminopeptidase N is a Major Receptor for the Entero-Pathogenic Coronavirus TGEV. Nature (1992) 357(6377):417–20. doi: 10.1038/357417a0 PMC7095137 1350661

[67] Peng JY Shin DL Li G Wu NH Herrler G. Time-Dependent Viral Interference Between Influenza Virus and Coronavirus in the Infection of Differentiated Porcine Airway Epithelial Cells. Virulence (2021) 12(1):1111–21. doi: 10.1080/21505594.2021.1911148 PMC8162253 34034617

[68] V’kovski P Kratzel A Steiner S Stalder H Thiel V. Coronavirus Biology and Replication: Implications for SARS-Cov-2. Nat Rev Microbiol (2021) 19(3):155–70. doi: 10.1038/s41579-020-00468-6 PMC7592455 33116300

[69] Kuo L Godeke GJ Raamsman MJ Masters PS Rottier PJ. Retargeting of Coronavirus by Substitution of the Spike Glycoprotein Ectodomain: Crossing the Host Cell Species Barrier. J Virol (2000) 74(3):1393–406. doi: 10.1128/JVI.74.3.1393-1406.2000 PMC111474 10627550

[70] Casais R Dove B Cavanagh D Britton P. Recombinant Avian Infectious Bronchitis Virus Expressing a Heterologous Spike Gene Demonstrates That the Spike Protein is a Determinant of Cell Tropism. J Virol (2003) 77(16):9084–9. doi: 10.1128/JVI.77.16.9084-9089.2003 PMC167237 12885925

[71] Armesto M Evans S Cavanagh D Abu-Median AB Keep S Britton P. A Recombinant Avian Infectious Bronchitis Virus Expressing a Heterologous Spike Gene Belonging to the 4/91 Serotype. PloS One (2011) 6(8):e24352. doi: 10.1371/journal.pone.0024352 21912629 PMC3166170

[72] Li W Zhang C Sui J Kuhn JH Moore MJ Luo S. Receptor and Viral Determinants of SARS-Coronavirus Adaptation to Human ACE2. EMBO J (2005) 24(8):1634–43. doi: 10.1038/sj.emboj.7600640 PMC1142572 15791205

[73] Paul PS Vaughn EM Halbur PG. Pathogenicity and Sequence Analysis Studies Suggest Potential Role of Gene 3 in Virulence of Swine Enteric and Respiratory Coronaviruses. Adv Exp Med Biol (1997) 412:317–21. doi: 10.1007/978-1-4899-1828-4_52 9192036

[74] Wesley RD Woods RD Cheung AK. Genetic Basis for the Pathogenesis of Transmissible Gastroenteritis Virus. J Virol (1990) 64(10):4761–6. doi: 10.1128/jvi.64.10.4761-4766.1990 PMC247963 2168963

[75] Sola I Alonso S Zúñiga S Balasch M Plana-Durán J Enjuanes L. Engineering the Transmissible Gastroenteritis Virus Genome as an Expression Vector Inducing Lactogenic Immunity. J Virol (2003) 77(7):4357–69. doi: 10.1128/JVI.77.7.4357-4369.2003 PMC150661 12634392

[76] Page KW Britton P Boursnell ME. Sequence Analysis of the Leader RNA of Two Porcine Coronaviruses: Transmissible Gastroenteritis Virus and Porcine Respiratory Coronavirus. Virus Genes (1990) 4(4):289–301. doi: 10.1007/BF00570024 1962975 PMC7088910

[77] Sola I Moreno JL Zúñiga S Alonso S Enjuanes L. Role of Nucleotides Immediately Flanking the Transcription-Regulating Sequence Core in Coronavirus Subgenomic Mrna Synthesis. J Virol (2005) 79(4):2506–16. doi: 10.1128/JVI.79.4.2506-2516.2005 PMC546574 15681451

[78] Bentley K Keep SM Armesto M Britton P. Identification of a Noncanonically Transcribed Subgenomic Mrna of Infectious Bronchitis Virus and Other Gammacoronaviruses. J Virol (2013) 87(4):2128–36. doi: 10.1128/JVI.02967-12 PMC3571483 23221558

[79] Sawicki SG Sawicki DL. Coronaviruses Use Discontinuous Extension for Synthesis of Subgenome-Length Negative Strands. Adv Exp Med Biol (1995) 380:499–506. doi: 10.1007/978-1-4615-1899-0_79 8830530

[80] Seow J Graham C Merrick B Acors S Pickering S Steel KJA. Longitudinal Observation and Decline of Neutralizing Antibody Responses in the Three Months Following SARS-CoV-2 Infection in Humans. Nat Microbiol (2020) 5(12):1598–607. doi: 10.1038/s41564-020-00813-8 PMC7610833 33106674

[81] Kaneko N Kuo HH Boucau J Farmer JR Allard-Chamard H Mahajan VS. Loss of Bcl-6-Expressing T Follicular Helper Cells and Germinal Centers in COVID-19. Cell (2020) 183(1):143–57.e13. doi: 10.1016/j.cell.2020.08.025 32877699 PMC7437499

[82] Long Q-X Tang X-J Shi Q-L Li Q Deng H-J Yuan J. Clinical and Immunological Assessment of Asymptomatic SARS-CoV-2 Infections. Nat Med (2020) 26(8):1200–4. doi: 10.1038/s41591-020-0965-6 32555424

[83] VanCott JL Brim TA Simkins RA Saif LJ. Isotype-Specific Antibody-Secreting Cells to Transmissible Gastroenteritis Virus and Porcine Respiratory Coronavirus in Gut- and Bronchus-Associated Lymphoid Tissues of Suckling Pigs. J Immunol (1993) 150(9):3990–4000. 8386204

[84] Brim TA VanCott JL Lunney JK Saif LJ. Lymphocyte Proliferation Responses of Pigs Inoculated With Transmissible Gastroenteritis Virus or Porcine Respiratory Coronavirus. Am J Vet Res (1994) 55(4):494–501. 8017695

[85] Wesley RD Woods RD. Induction of Protective Immunity Against Transmissible Gastroenteritis Virus After Exposure of Neonatal Pigs to Porcine Respiratory Coronavirus. Am J Vet Res (1996) 57(2):157–62. 8633800

[86] Cox E Pensaert MB Callebaut P. Intestinal Protection Against Challenge With Transmissible Gastroenteritis Virus of Pigs Immune After Infection With the Porcine Respiratory Coronavirus. Vaccine (1993) 11(2):267–72. doi: 10.1016/0264-410X(93)90028-V PMC7131394 8382421

[87] VanCott JL Brim TA Lunney JK Saif LJ. Contribution of Antibody-Secreting Cells Induced in Mucosal Lymphoid Tissues of Pigs Inoculated With Respiratory or Enteric Strains of Coronavirus to Immunity Against Enteric Coronavirus Challenge. J Immunol (1994) 152(8):3980–90. 8144965

[88] Swadling L Diniz MO Schmidt NM Amin OE Chandran A Shaw E. Pre-Existing Polymerase-Specific T Cells Expand in Abortive Seronegative SARS-CoV-2. Nature (2022) 601(7891):110–7. doi: 10.1038/s41586-021-04186-8 PMC8732273 34758478

[89] Peng Y Felce SL Dong D Penkava F Mentzer AJ Yao X. An Immunodominant NP(105-113)-B*07:02 Cytotoxic T Cell Response Controls Viral Replication and is Associated With Less Severe COVID-19 Disease. Nat Immunol (2022) 23(1):50–61. doi: 10.1038/s41590-021-01084-z 34853448 PMC8709787

