# Supplementary material for: Porcine Respiratory Coronavirus as a Model for Acute Respiratory Coronavirus Disease
Source: Front Immunol. 2022 Mar 28;13:867707. doi: 10.3389/fimmu.2022.867707 (PMC8995773; doi:10.3389/fimmu.2022.867707)
Supplement: Supplementary file 1 [file DataSheet_1.docx]

Supplementary Material

# Supplementary figures and tables:


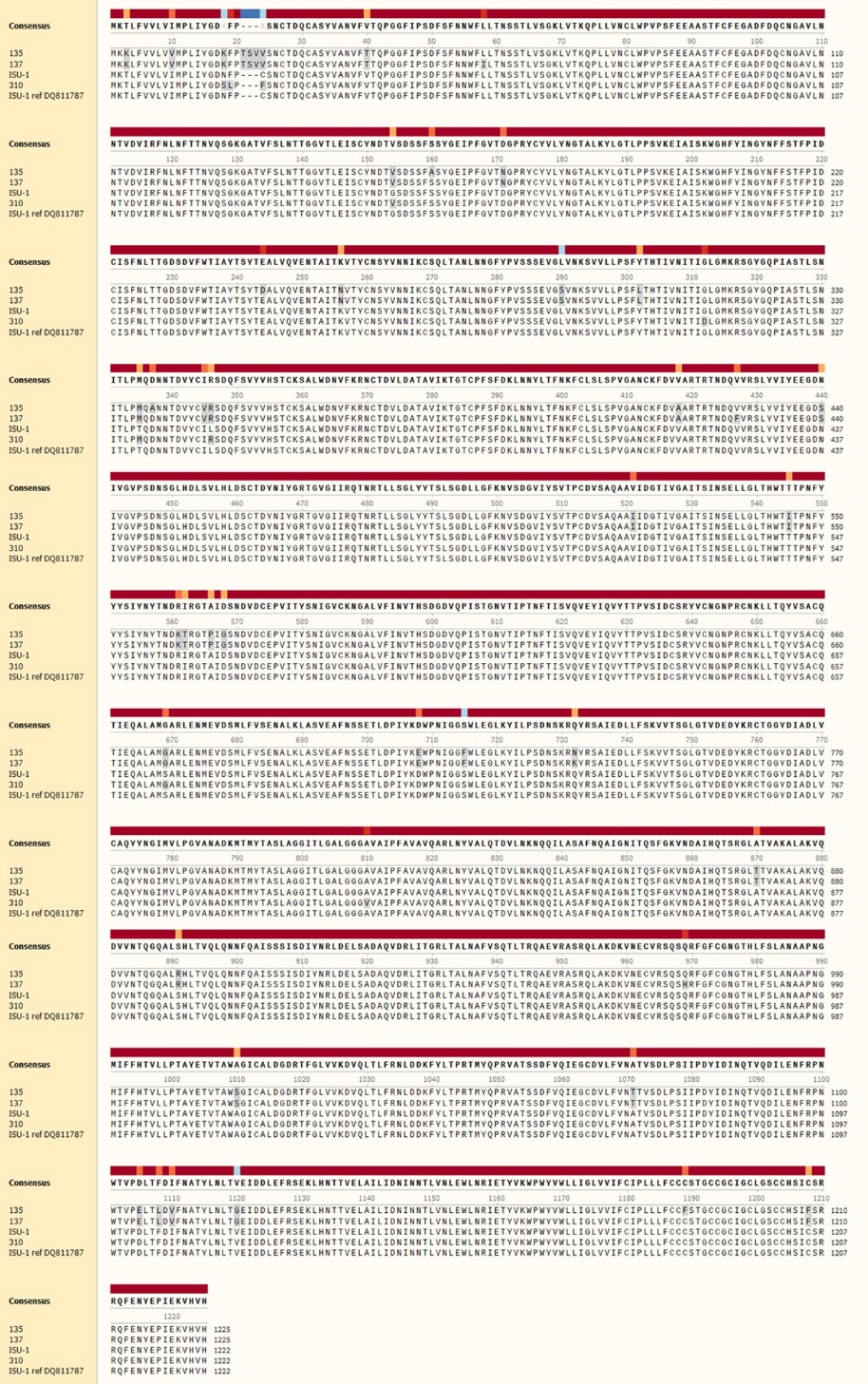


**Supplementary Figure 1: Differences in the amino acid sequence of the S glycoprotein.** Nucleotides encoding the S glycoprotein, position 20349 – 24026 for 135, 20349 – 24026 for 137, 20342 – 24011 to ISU-1, 20342 – 24010 for 310 and 20345 – 24013 for the ISU- 1 reference sequence (DQ811787) were aligned using MAFFT in Snapgene. The alignment was subsequently translated in Snapgene. Differences in comparison to the ISU-1 reference sequence are highlighted in grey.


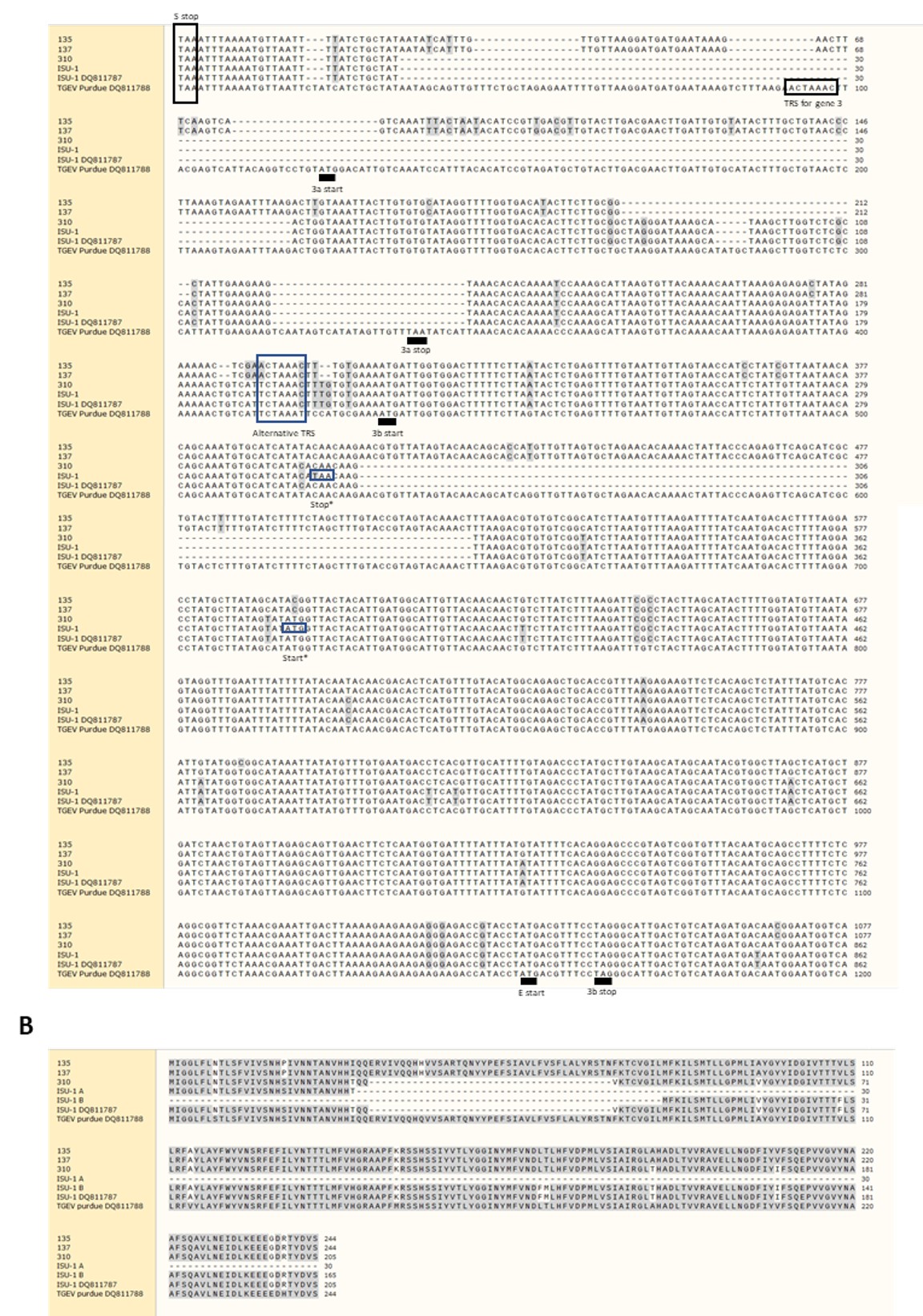


**Supplementary Figure 2: PRCV strains 135 and 137 potentially encode a full-length accessory protein 3b.** (A) Nucleotides from position 24024 – 25100 for 135 and 137, 24008 – 24869 for 310, 24009 – 24870 for ISU-1 were aligned using MAFFT in Snapgene to positions 24011 – 24872 of the ISU- 1 reference sequence (DQ811787) and 25029 – 25894 of the TGEV Purdue sequence (DQ811788). Stop and start codon are underlined and TRS sequences highlighted in the boxed areas. Start and stop codon highlighted with a * are unique to ISU-1. (B) Amino acid alignment of the residues encoding open reading frame 3a and 3b. ISU-1 encodes potentially two ORFs, highlighted as A and B. (A and B) Differences in grey are highlighted in comparison to the TGEV sequence.


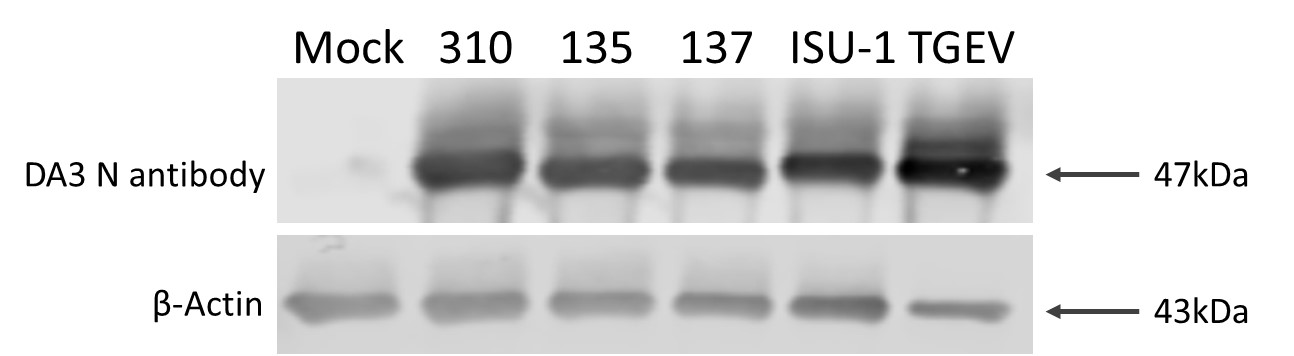


**Supplementary Figure 3: DA3 antibody against the N protein is cross reactive between PRCV strains.** ST cells were inoculated with 10^4^ PFU of either PRCV 310, 135, 137 and ISU-1, TGEV 772/70 or mock inoculated with medium only. Cell lysates were harvested at 24 hpi and separated on a 4 -15% polyacrylamide gel and N protein detected an DA3 antibody. Anti- β actin was used as a loading control. Two bands were detected 43kDa, relating to β Actin and 47kDa relating to the N protein.


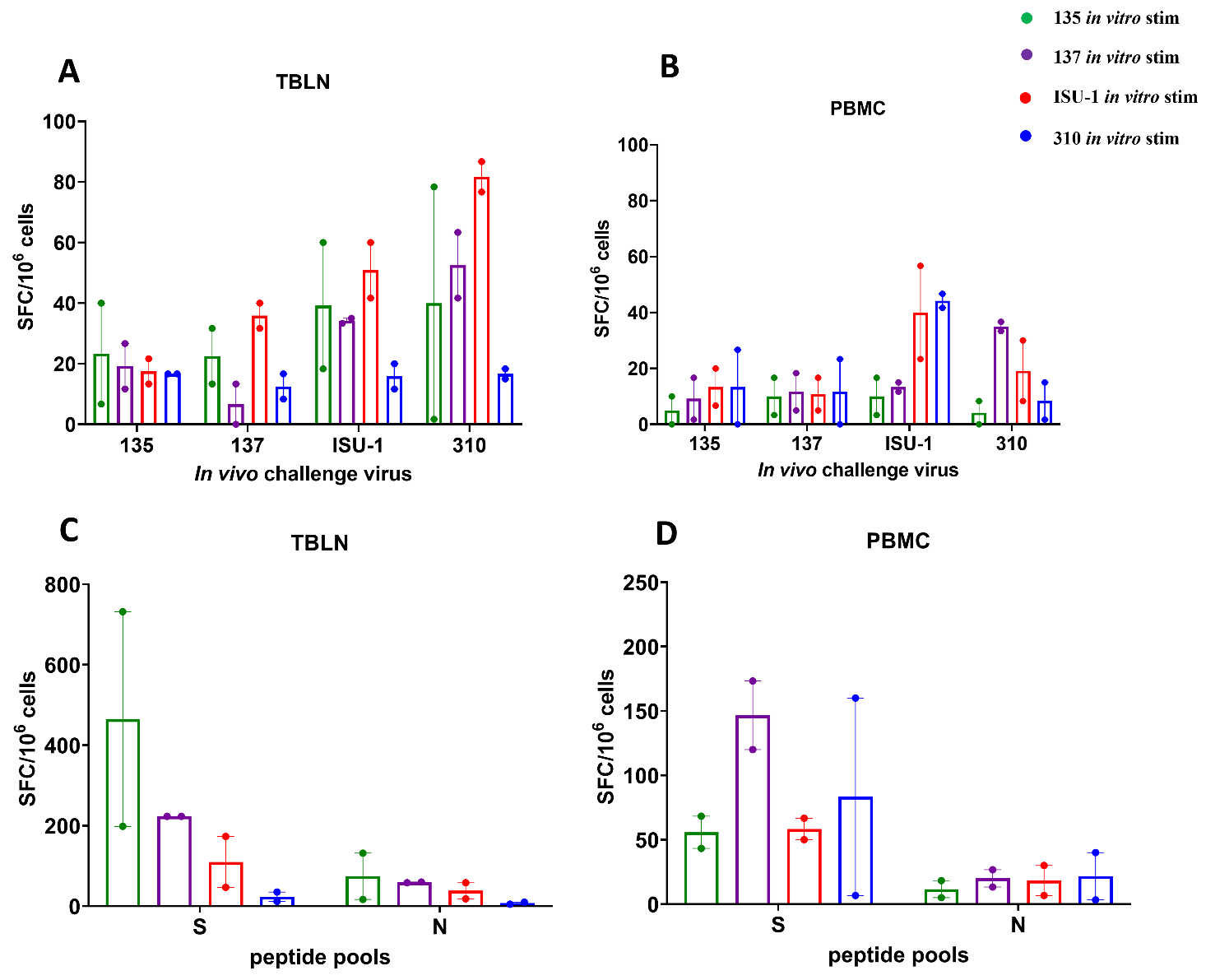


**Supplementary Figure 4. IFNγ ELISpot responses in TBLN and PBMC from pigs challenged with PRCV strains 135, 137, 310 and ISU-1.** IFNγ secreting spot forming cells (SFC) were enumerated in tracheobronchial lymph nodes (TBLN) and PBMC at 5 DPI, following stimulation with live PRCV strains 135, 137, ISU-1 or 310 (A and B) or overlapping pool of peptides covering S and N proteins (C and D). Each symbol represents an individual animal, the top of the bar the mean and the line the standard error (SEM).


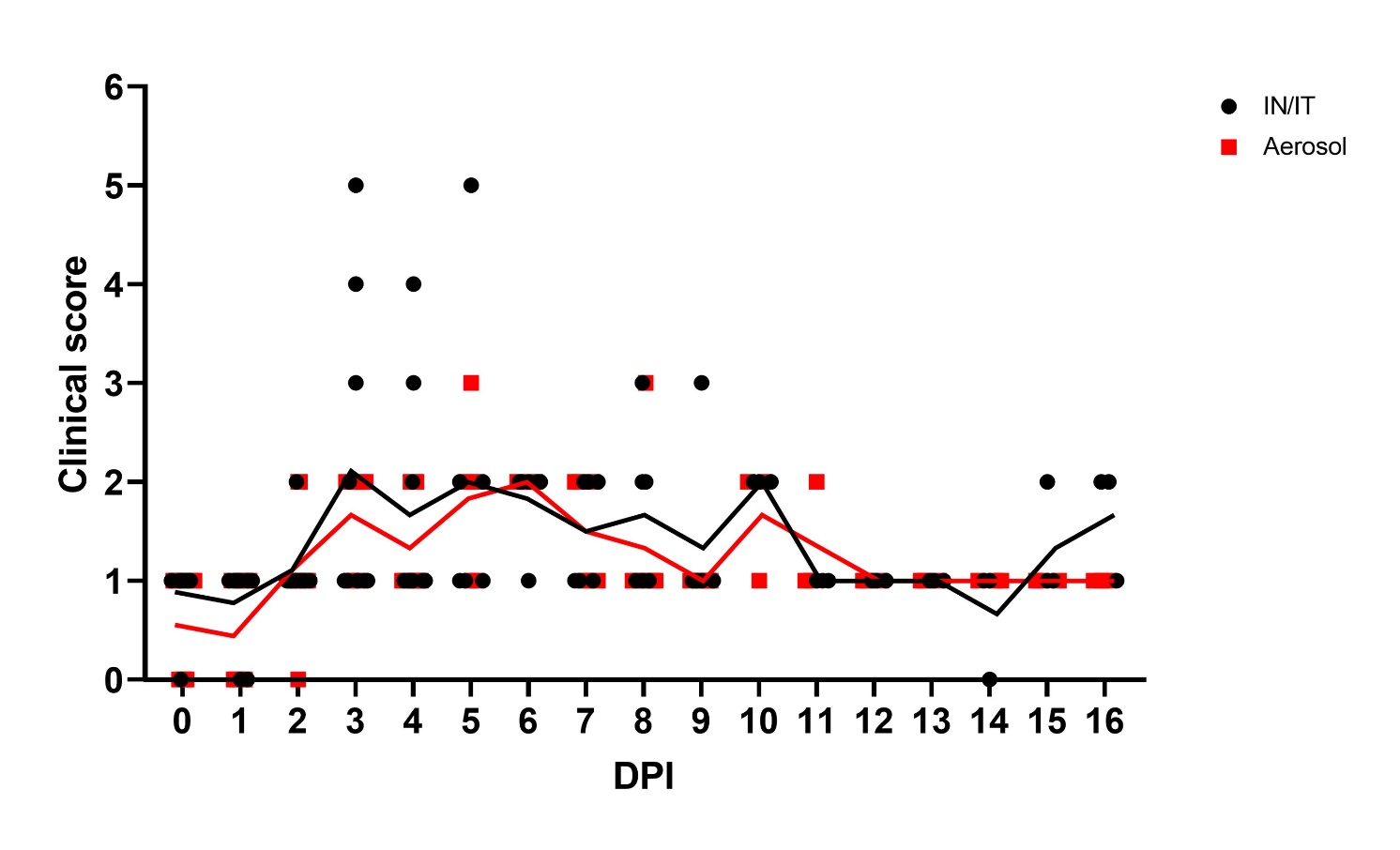


**Supplementary Figure 5: Clinical signs after *in vivo* challenge with PRCV 135.** Nine pigs per group were inoculated with 1 x 10^7^ PFU of 135 by the IT/IN (black) or by Aer (red) route. Three animals from each group were culled on 4, 9 and 16 DPI. Clinical signs including demeanour, appetite, respiratory signs, sneezing, coughing, nasal and eye discharge, faeces consistency and rectal temperature were assessed and the clinical score displayed. Clinical scores are listed in Supp Table 1.


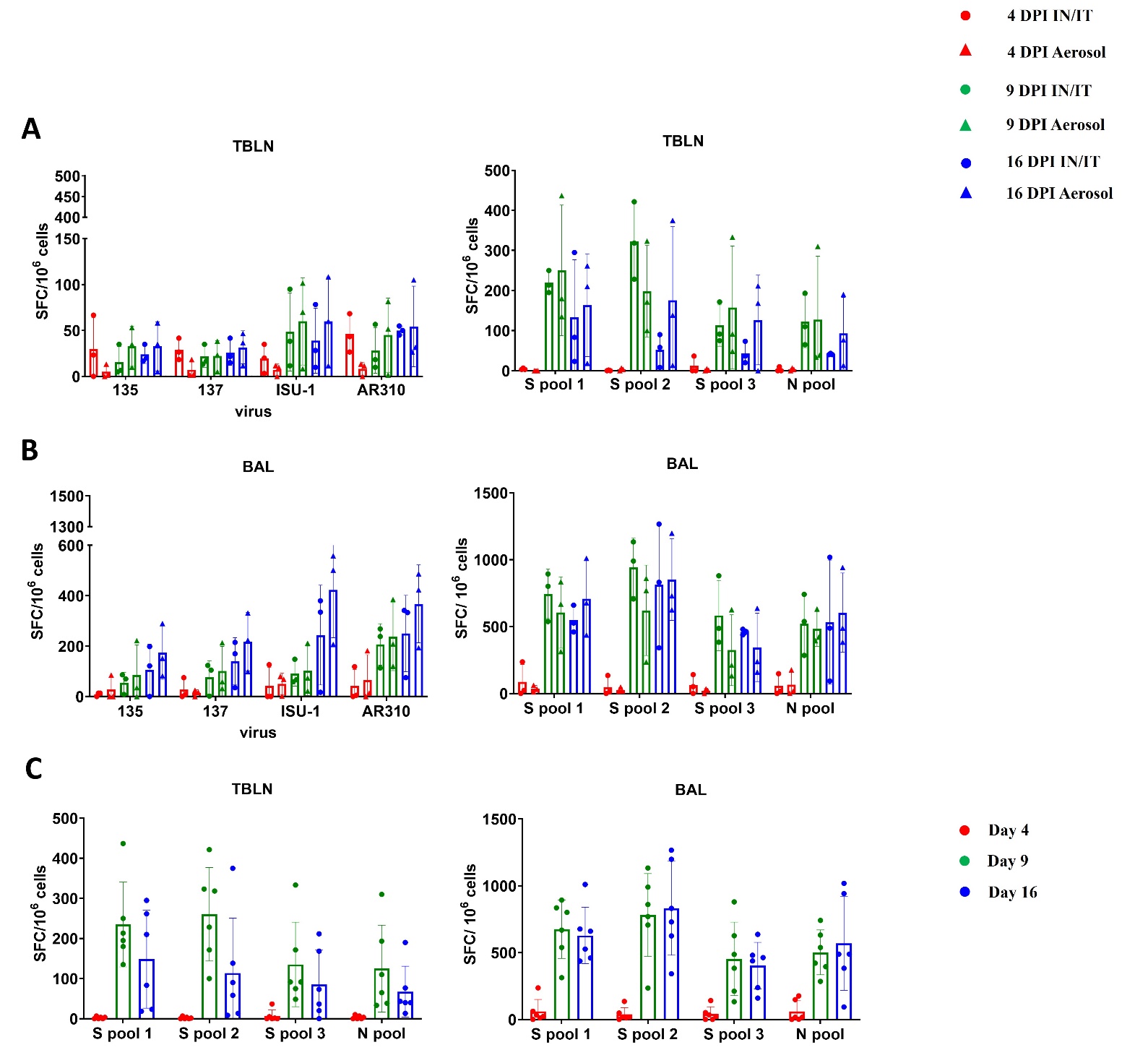


**Supplementary Figure 6. IFNγ ELISpot responses in TBLN and BAL after *in vivo* challenge with 135 strain.** IFNγ secreting spot forming cells (SFC) were enumerated in tracheobronchial lymph nodes (TBLN) **(A)** and bronchoalveolar lavage (BAL) **(B)** at 4, 9 and 16 DPI, following stimulation with 135, 137, ISU-1 or 310 strains or pools of peptides covering S and N proteins (individual S peptide pools 1, 2 and 3 are shown). For each cull day IN/IT and Aer groups are displayed separately. Individual peptide pools grouped by cull day, irrespective of route of administration are also shown **(C)**. Each symbol represents an individual animal, the top of the bar the mean and the line the standard deviation (SD).


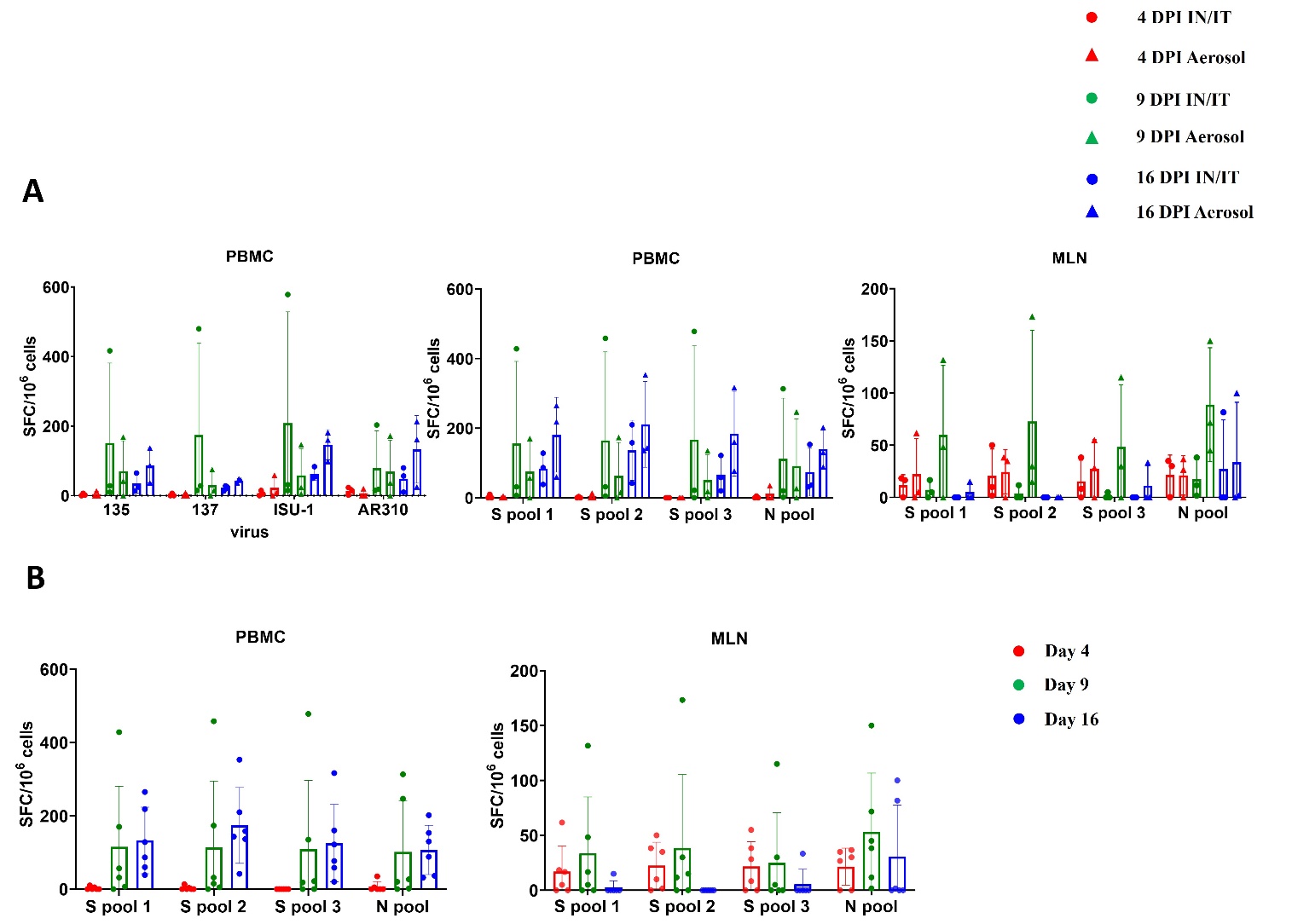


**Supplementary Figure 7. IFNγ ELISpot responses in PBMC and MLN after *in vivo* challenge with PRCV 135.** IFNγ secreting spot forming cells (SFC) were enumerated in **(A)** peripheral blood mononuclear cells (PBMC) and **(B)** mesenteric lymph nodes (MLN) at 4, 9 and 16 DPI, following stimulation with 135, 137, ISU-1 or 310 viruses or pool of peptides covering S and N proteins (individual S peptide pools 1, 2 and 3 are shown). For each cull day IN/IT and Aer groups are displayed separately. Individual peptide pools grouped by cull day, irrespective of route of administration are also shown (**C)**. Each symbol represents an individual animal, the top of the bar the mean and the line the standard deviation (SD).


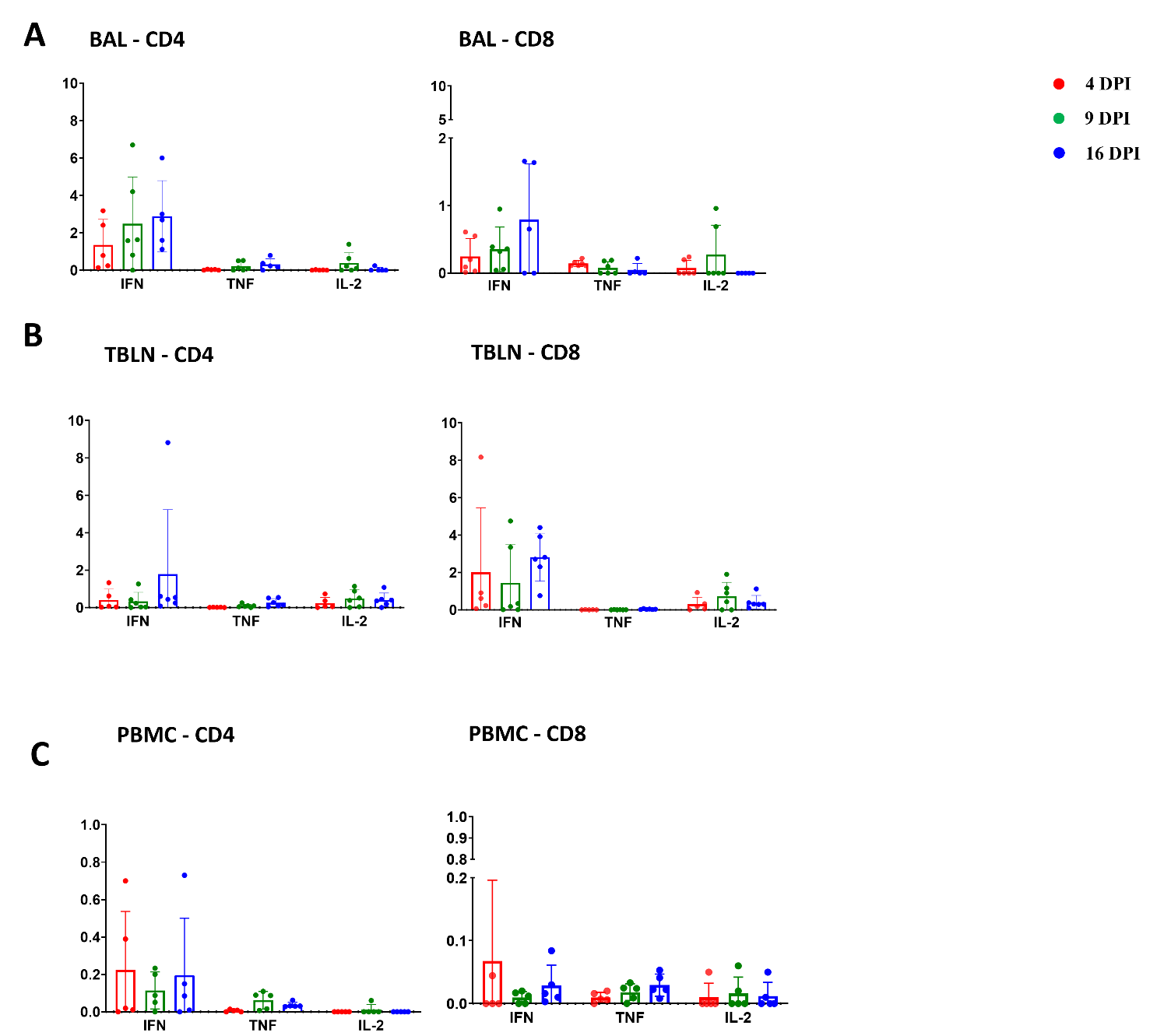


**Supplementary Figure 8. T cell cytokine responses in BAL, TBLN and PBMC.** BAL **(A)**, TBLN **(B)** and PBMC **(C)** were collected at 4, 9 and 16 DPI. Cells were stimulated with 135 virus and IFNγ, TNF and IL-2 cytokine secretion was measured in CD4 and CD8 T cells by intracellular cytokine staining. Each symbol represents an individual animal, the top of the bar the mean and the line the standard deviation (SD).

**Supplementary Table 1. Clinical signs in pigs.** Pigs were scored daily for the signs below and scores from all 7 parameters were added to give a maximum of 19.

|  | ***Parameter*** | ***Criteria*** | ***Score*** |
| --- | --- | --- | --- |
| **1** | **Alertness** | Attentive (curious, alert) | **0** |
|  |  | Slightly reduced – hesitant, disinterested | **1** |
|  |  | Inactive, gets up only when stimulated, lies down again | **2** |
|  |  | Recumbent, won’t get up when stimulated | **3** |
|  | | |  |
| **2** | **Body shape / posture** | Relaxed, full stomach, ‘round’ body | **0** |
|  |  | Hunched back, empty stomach, thinned body muscles | **2** |
|  |  | Flaccid or Hunched & rigid body, emaciated, ribs & backbone showing | **3** |
|  | | |  |
| **3** | **Breathing** | Normal | **0** |
|  |  | Sneezing / coughing | **1** |
|  |  | Intermittent breathing difficulties, or increase/decrease respiratory rate | **2** |
|  |  | Persistent breathing distress, wheezing | **3** |
|  | | | |
| **4** | **Eyes/Nose** | Eyes bright and clear, light pink conjunctiva | **0** |
|  |  | Clear discharge from eyes and/or nose, reddened eyes | **1** |
|  |  | Yellow / green discharge from eyes and/or nose | **2** |
|  | | |  |
| **5** | **Defecation** | Soft faeces, normal amount | **0** |
|  |  | Loose faeces | **1** |
|  |  | Diarrhoea | **2** |
|  | | |  |
| **6** | **Appetite** | Greedy, hungry, all food finished | **0** |
|  |  | Eats slowly when fed, some food remaining | **1** |
|  |  | Does not eat when fed but tastes food. Food only partially eaten. | **2** |
|  |  | Shows no interest in food, nothing eaten/drunk. | **3** |
|  | | |  |
| **7** | **Temperature** | 37.0^o^C – 38.9^o^C | **0** |
|  |  | 39.0^o^C – 39.9^o^C or less than 37^o^C | **1** |
|  |  | 40.0^o^C – 40.9^o^C | **2** |
|  |  | 41.0^o^C or above | **3** |

**Supplementary Table 2. Peptide pools covering the spike of PRCV 135 virus (16-mer offset by 4).**

| **S1 – S100 = Pool 1, S101 – S200 = Pool 2, S201 – S305 = Pool 3**   \| S.1 \| MKKLFVVLVVMPLIYG \|  \| S.63 \| VENTAITNVTYCNSYV \|  \| S.125 \| LGFKNVSDGVIYSVTP \| \| --- \| --- \| --- \| --- \| --- \| --- \| --- \| --- \| \| S.2 \| FVVLVVMPLIYGDKFP \|  \| S.64 \| AITNVTYCNSYVNNIK \|  \| S.126 \| NVSDGVIYSVTPCDVS \| \| S.3 \| VVMPLIYGDKFPTSVV \|  \| S.65 \| VTYCNSYVNNIKCSQL \|  \| S.127 \| GVIYSVTPCDVSAQAA \| \| S.4 \| LIYGDKFPTSVVSNCT \|  \| S.66 \| NSYVNNIKCSQLTANL \|  \| S.128 \| SVTPCDVSAQAAIIDG \| \| S.5 \| DKFPTSVVSNCTDQCA \|  \| S.67 \| NNIKCSQLTANLNNGF \|  \| S.129 \| CDVSAQAAIIDGTIVG \| \| S.6 \| TSVVSNCTDQCASYVA \|  \| S.68 \| CSQLTANLNNGFYPVS \|  \| S.130 \| AQAAIIDGTIVGAITS \| \| S.7 \| SNCTDQCASYVANVFT \|  \| S.69 \| TANLNNGFYPVSSSEV \|  \| S.131 \| IIDGTIVGAITSINSE \| \| S.8 \| DQCASYVANVFTTQPG \|  \| S.70 \| NNGFYPVSSSEVGSVN \|  \| S.132 \| TIVGAITSINSELLGL \| \| S.9 \| SYVANVFTTQPGGFIP \|  \| S.71 \| YPVSSSEVGSVNKSVV \|  \| S.133 \| AITSINSELLGLTHWT \| \| S.10 \| NVFTTQPGGFIPSDFS \|  \| S.72 \| SSEVGSVNKSVVLLPS \|  \| S.134 \| INSELLGLTHWTITPN \| \| S.11 \| TQPGGFIPSDFSFNNW \|  \| S.73 \| GSVNKSVVLLPSFLTH \|  \| S.135 \| LLGLTHWTITPNFYYY \| \| S.12 \| GFIPSDFSFNNWFLLT \|  \| S.74 \| KSVVLLPSFLTHTIVN \|  \| S.136 \| THWTITPNFYYYSIYN \| \| S.13 \| SDFSFNNWFLLTNSST \|  \| S.75 \| LLPSFLTHTIVNITIG \|  \| S.137 \| ITPNFYYYSIYNYTND \| \| S.14 \| FNNWFLLTNSSTLVSG \|  \| S.76 \| FLTHTIVNITIGLGMK \|  \| S.138 \| FYYYSIYNYTNDKTRG \| \| S.15 \| FLLTNSSTLVSGKLVT \|  \| S.77 \| TIVNITIGLGMKRSGY \|  \| S.139 \| SIYNYTNDKTRGTPIG \| \| S.16 \| NSSTLVSGKLVTKQPL \|  \| S.78 \| ITIGLGMKRSGYGQPI \|  \| S.140 \| YTNDKTRGTPIGSNDV \| \| S.17 \| LVSGKLVTKQPLLVNC \|  \| S.79 \| LGMKRSGYGQPIASTL \|  \| S.141 \| KTRGTPIGSNDVDCEP \| \| S.18 \| KLVTKQPLLVNCLWPV \|  \| S.80 \| RSGYGQPIASTLSNIT \|  \| S.142 \| TPIGSNDVDCEPVITY \| \| S.19 \| KQPLLVNCLWPVPSFE \|  \| S.81 \| GQPIASTLSNITLPMQ \|  \| S.143 \| SNDVDCEPVITYSNIG \| \| S.20 \| LVNCLWPVPSFEEAAS \|  \| S.82 \| ASTLSNITLPMQANNT \|  \| S.144 \| DCEPVITYSNIGVCKN \| \| S.21 \| LWPVPSFEEAASTFCF \|  \| S.83 \| SNITLPMQANNTDVYC \|  \| S.145 \| VITYSNIGVCKNGALV \| \| S.22 \| PSFEEAASTFCFEGAD \|  \| S.84 \| LPMQANNTDVYCVRSD \|  \| S.146 \| SNIGVCKNGALVFINV \| \| S.23 \| EAASTFCFEGADFDQC \|  \| S.85 \| ANNTDVYCVRSDQFSV \|  \| S.147 \| VCKNGALVFINVTHSD \| \| S.24 \| TFCFEGADFDQCNGAV \|  \| S.86 \| DVYCVRSDQFSVYVHS \|  \| S.148 \| GALVFINVTHSDGDVQ \| \| S.25 \| EGADFDQCNGAVLNNT \|  \| S.87 \| VRSDQFSVYVHSTCKS \|  \| S.149 \| FINVTHSDGDVQPIST \| \| S.26 \| FDQCNGAVLNNTVDVI \|  \| S.88 \| QFSVYVHSTCKSALWD \|  \| S.150 \| THSDGDVQPISTGNVT \| \| S.27 \| NGAVLNNTVDVIRFNL \|  \| S.89 \| YVHSTCKSALWDNVFK \|  \| S.151 \| GDVQPISTGNVTIPTN \| \| S.28 \| LNNTVDVIRFNLNFTT \|  \| S.90 \| TCKSALWDNVFKRNCT \|  \| S.152 \| PISTGNVTIPTNFTIS \| \| S.29 \| VDVIRFNLNFTTNVQS \|  \| S.91 \| ALWDNVFKRNCTDVLD \|  \| S.153 \| GNVTIPTNFTISVQVE \| \| S.30 \| RFNLNFTTNVQSGKGA \|  \| S.92 \| NVFKRNCTDVLDATAV \|  \| S.154 \| IPTNFTISVQVEYIQV \| \| S.31 \| NFTTNVQSGKGATVFS \|  \| S.93 \| RNCTDVLDATAVIKTG \|  \| S.155 \| FTISVQVEYIQVYTTP \| \| S.32 \| NVQSGKGATVFSLNTT \|  \| S.94 \| DVLDATAVIKTGTCPF \|  \| S.156 \| VQVEYIQVYTTPVSID \| \| S.33 \| GKGATVFSLNTTGGVT \|  \| S.95 \| ATAVIKTGTCPFSFDK \|  \| S.157 \| YIQVYTTPVSIDCSRY \| \| S.34 \| TVFSLNTTGGVTLEIS \|  \| S.96 \| IKTGTCPFSFDKLNNY \|  \| S.158 \| YTTPVSIDCSRYVCNG \| \| S.35 \| LNTTGGVTLEISCYND \|  \| S.97 \| TCPFSFDKLNNYLTFN \|  \| S.159 \| VSIDCSRYVCNGNPRC \| \| S.36 \| GGVTLEISCYNDTVSD \|  \| S.98 \| SFDKLNNYLTFNKFCL \|  \| S.160 \| CSRYVCNGNPRCNKLL \| \| S.37 \| LEISCYNDTVSDSSFA \|  \| S.99 \| LNNYLTFNKFCLSLSP \|  \| S.161 \| VCNGNPRCNKLLTQYV \| \| S.38 \| CYNDTVSDSSFASYGE \|  \| S.100 \| LTFNKFCLSLSPVGAN \|  \| S.162 \| NPRCNKLLTQYVSACQ \| \| S.39 \| TVSDSSFASYGEIPFG \|  \| S.101 \| KFCLSLSPVGANCKFD \|  \| S.163 \| NKLLTQYVSACQTIEQ \| \| S.40 \| SSFASYGEIPFGVTNG \|  \| S.102 \| SLSPVGANCKFDVAAR \|  \| S.164 \| TQYVSACQTIEQALAM \| \| S.41 \| SYGEIPFGVTNGPRYC \|  \| S.103 \| VGANCKFDVAARTRTN \|  \| S.165 \| SACQTIEQALAMGARL \| \| S.42 \| IPFGVTNGPRYCYVLY \|  \| S.104 \| CKFDVAARTRTNDQVV \|  \| S.166 \| TIEQALAMGARLENME \| \| S.43 \| VTNGPRYCYVLYNGTA \|  \| S.105 \| VAARTRTNDQVVRSLY \|  \| S.167 \| ALAMGARLENMEVDSM \| \| S.44 \| PRYCYVLYNGTALKYL \|  \| S.106 \| TRTNDQVVRSLYVIYE \|  \| S.168 \| GARLENMEVDSMLFVS \| \| S.45 \| YVLYNGTALKYLGTLP \|  \| S.107 \| DQVVRSLYVIYEEGDS \|  \| S.169 \| ENMEVDSMLFVSENAL \| \| S.46 \| NGTALKYLGTLPPSVK \|  \| S.108 \| RSLYVIYEEGDSIVGV \|  \| S.170 \| VDSMLFVSENALKLAS \| \| S.47 \| LKYLGTLPPSVKEIAI \|  \| S.109 \| VIYEEGDSIVGVPSDN \|  \| S.171 \| LFVSENALKLASVEAF \| \| S.48 \| GTLPPSVKEIAISKWG \|  \| S.110 \| EGDSIVGVPSDNSGLH \|  \| S.172 \| ENALKLASVEAFNSSE \| \| S.49 \| PSVKEIAISKWGHFYI \|  \| S.111 \| IVGVPSDNSGLHDLSV \|  \| S.173 \| KLASVEAFNSSETLDP \| \| S.50 \| EIAISKWGHFYINGYN \|  \| S.112 \| PSDNSGLHDLSVLHLD \|  \| S.174 \| VEAFNSSETLDPIYKE \| \| S.51 \| SKWGHFYINGYNFFST \|  \| S.113 \| SGLHDLSVLHLDSCTD \|  \| S.175 \| NSSETLDPIYKEWPNI \| \| S.52 \| HFYINGYNFFSTFPID \|  \| S.114 \| DLSVLHLDSCTDYNIY \|  \| S.176 \| TLDPIYKEWPNIGGFW \| \| S.53 \| NGYNFFSTFPIDCISF \|  \| S.115 \| LHLDSCTDYNIYGRTG \|  \| S.177 \| IYKEWPNIGGFWLEGL \| \| S.54 \| FFSTFPIDCISFNLTT \|  \| S.116 \| SCTDYNIYGRTGVGII \|  \| S.178 \| WPNIGGFWLEGLKYIL \| \| S.55 \| FPIDCISFNLTTGDSD \|  \| S.117 \| YNIYGRTGVGIIRQTN \|  \| S.179 \| GGFWLEGLKYILPSDN \| \| S.56 \| CISFNLTTGDSDVFWT \|  \| S.118 \| GRTGVGIIRQTNRTLL \|  \| S.180 \| LEGLKYILPSDNSKRN \| \| S.57 \| NLTTGDSDVFWTIAYT \|  \| S.119 \| VGIIRQTNRTLLSGLY \|  \| S.181 \| KYILPSDNSKRNYRSA \| \| S.58 \| GDSDVFWTIAYTSYTD \|  \| S.120 \| RQTNRTLLSGLYYTSL \|  \| S.182 \| PSDNSKRNYRSAIEDL \| \| S.59 \| VFWTIAYTSYTDALVQ \|  \| S.121 \| RTLLSGLYYTSLSGDL \|  \| S.183 \| SKRNYRSAIEDLLFSK \| \| S.60 \| IAYTSYTDALVQVENT \|  \| S.122 \| SGLYYTSLSGDLLGFK \|  \| S.184 \| YRSAIEDLLFSKVVTS \| \| S.61 \| SYTDALVQVENTAITN \|  \| S.123 \| YTSLSGDLLGFKNVSD \|  \| S.185 \| IEDLLFSKVVTSGLGT \| \| S.62 \| ALVQVENTAITNVTYC \|  \| S.124 \| SGDLLGFKNVSDGVIY \|  \| S.186 \| LFSKVVTSGLGTVDED \| \| S.187 \| VVTSGLGTVDEDYKRC \|  \| S.255 \| GDRTFGLVVKDVQLTL \|  \| \| S.188 \| GLGTVDEDYKRCTGGY \|  \| S.256 \| FGLVVKDVQLTLFRNL \|  \| \| S.189 \| VDEDYKRCTGGYDIAD \|  \| S.257 \| VKDVQLTLFRNLDDKF \|  \| \| S.190 \| YKRCTGGYDIADLVCA \|  \| S.258 \| QLTLFRNLDDKFYLTP \|  \| \| S.191 \| TGGYDIADLVCAQYYN \|  \| S.259 \| FRNLDDKFYLTPRTMY \|  \| \| S.192 \| DIADLVCAQYYNGIMV \|  \| S.260 \| DDKFYLTPRTMYQPRV \|  \| \| S.193 \| LVCAQYYNGIMVLPGV \|  \| S.261 \| YLTPRTMYQPRVATSS \|  \| \| S.194 \| QYYNGIMVLPGVANAD \|  \| S.262 \| RTMYQPRVATSSDFVQ \|  \| \| S.195 \| GIMVLPGVANADKMTM \|  \| S.263 \| QPRVATSSDFVQIEGC \|  \| \| S.196 \| LPGVANADKMTMYTAS \|  \| S.264 \| ATSSDFVQIEGCDVLF \|  \| \| S.197 \| ANADKMTMYTASLAGG \|  \| S.265 \| DFVQIEGCDVLFVNTT \|  \| \| S.198 \| KMTMYTASLAGGITLG \|  \| S.266 \| IEGCDVLFVNTTVSDL \|  \| \| S.199 \| YTASLAGGITLGALGG \|  \| S.267 \| DVLFVNTTVSDLPSII \|  \| \| S.200 \| LAGGITLGALGGGAVA \|  \| S.268 \| VNTTVSDLPSIIPDYI \|  \| \| S.201 \| ITLGALGGGAVAIPFA \|  \| S.269 \| VSDLPSIIPDYIDINQ \|  \| \| S.202 \| ALGGGAVAIPFAVAVQ \|  \| S.270 \| PSIIPDYIDINQTVQD \|  \| \| S.203 \| GAVAIPFAVAVQARLN \|  \| S.271 \| PDYIDINQTVQDILEN \|  \| \| S.204 \| IPFAVAVQARLNYVAL \|  \| S.272 \| DINQTVQDILENFRPN \|  \| \| S.205 \| VAVQARLNYVALQTDV \|  \| S.273 \| TVQDILENFRPNWTVP \|  \| \| S.206 \| ARLNYVALQTDVLNKN \|  \| S.274 \| ILENFRPNWTVPELTL \|  \| \| S.207 \| YVALQTDVLNKNQQIL \|  \| S.275 \| FRPNWTVPELTLDVFN \|  \| \| S.208 \| QTDVLNKNQQILASAF \|  \| S.276 \| WTVPELTLDVFNATYL \|  \| \| S.209 \| LNKNQQILASAFNQAI \|  \| S.277 \| ELTLDVFNATYLNLTG \|  \| \| S.210 \| QQILASAFNQAIGNIT \|  \| S.278 \| DVFNATYLNLTGEIDD \|  \| \| S.211 \| ASAFNQAIGNITQSFG \|  \| S.279 \| ATYLNLTGEIDDLEFR \|  \| \| S.212 \| NQAIGNITQSFGKVND \|  \| S.280 \| NLTGEIDDLEFRSEKL \|  \| \| S.213 \| GNITQSFGKVNDAIHQ \|  \| S.281 \| EIDDLEFRSEKLHNTT \|  \| \| S.214 \| QSFGKVNDAIHQTSRG \|  \| S.282 \| LEFRSEKLHNTTVELA \|  \| \| S.215 \| KVNDAIHQTSRGLTTV \|  \| S.283 \| SEKLHNTTVELAILID \|  \| \| S.216 \| AIHQTSRGLTTVAKAL \|  \| S.284 \| HNTTVELAILIDNINN \|  \| \| S.217 \| TSRGLTTVAKALAKVQ \|  \| S.285 \| VELAILIDNINNTLVN \|  \| \| S.218 \| LTTVAKALAKVQDVVN \|  \| S.286 \| ILIDNINNTLVNLEWL \|  \| \| S.219 \| AKALAKVQDVVNTQGQ \|  \| S.287 \| NINNTLVNLEWLNRIE \|  \| \| S.220 \| AKVQDVVNTQGQALRH \|  \| S.288 \| TLVNLEWLNRIETYVK \|  \| \| S.221 \| DVVNTQGQALRHLTVQ \|  \| S.289 \| LEWLNRIETYVKWPWY \|  \| \| S.222 \| TQGQALRHLTVQLQNN \|  \| S.290 \| NRIETYVKWPWYVWLL \|  \| \| S.223 \| ALRHLTVQLQNNFQAI \|  \| S.291 \| TYVKWPWYVWLLIGLV \|  \| \| S.224 \| LTVQLQNNFQAISSSI \|  \| S.292 \| WPWYVWLLIGLVVIFC \|  \| \| S.225 \| LQNNFQAISSSISDIY \|  \| S.293 \| VWLLIGLVVIFCIPLL \|  \| \| S.226 \| FQAISSSISDIYNRLD \|  \| S.294 \| IGLVVIFCIPLLLFCC \|  \| \| S.227 \| SSSISDIYNRLDELSA \|  \| S.295 \| VIFCIPLLLFCCFSTG \|  \| \| S.228 \| SDIYNRLDELSADAQV \|  \| S.296 \| IPLLLFCCFSTGCCGC \|  \| \| S.229 \| NRLDELSADAQVDRLI \|  \| S.297 \| LFCCFSTGCCGCIGCL \|  \| \| S.230 \| ELSADAQVDRLITGRL \|  \| S.298 \| FSTGCCGCIGCLGSCC \|  \| \| S.231 \| DAQVDRLITGRLTALN \|  \| S.299 \| CCGCIGCLGSCCHSIF \|  \| \| S.232 \| DRLITGRLTALNAFVS \|  \| S.300 \| IGCLGSCCHSIFSRRQ \|  \| \| S.233 \| TGRLTALNAFVSQTLT \|  \| S.301 \| GSCCHSIFSRRQFENY \|  \| \| S.234 \| TALNAFVSQTLTRQAE \|  \| S.302 \| HSIFSRRQFENYEPIE \|  \| \| S.235 \| AFVSQTLTRQAEVRAS \|  \| S.303 \| SRRQFENYEPIEKVHV \|  \| \| S.236 \| QTLTRQAEVRASRQLA \|  \| S.304 \| FENYEPIEKVHVH \|  \| \| S.237 \| RQAEVRASRQLAKDKV \|  \| S.305 \| RRQFENYEPIEKVHVH \|  \| \| S.238 \| VRASRQLAKDKVNECV \|  \|  \|  \|  \| \| S.239 \| RQLAKDKVNECVRSQS \|  \|  \|  \|  \| \| S.240 \| KDKVNECVRSQSQRFG \|  \|  \|  \|  \| \| S.241 \| NECVRSQSQRFGFCGN \|  \|  \|  \|  \| \| S.242 \| RSQSQRFGFCGNGTHL \|  \|  \|  \|  \| \| S.243 \| QRFGFCGNGTHLFSLA \|  \|  \|  \|  \| \| S.244 \| FCGNGTHLFSLANAAP \|  \|  \|  \|  \| \| S.245 \| GTHLFSLANAAPNGMI \|  \|  \|  \|  \| \| S.246 \| FSLANAAPNGMIFFHT \|  \|  \|  \|  \| \| S.247 \| NAAPNGMIFFHTVLLP \|  \|  \|  \|  \| \| S.248 \| NGMIFFHTVLLPTAYE \|  \|  \|  \|  \| \| S.249 \| FFHTVLLPTAYETVTA \|  \|  \|  \|  \| \| S.250 \| VLLPTAYETVTAWSGI \|  \|  \|  \|  \| \| S.251 \| TAYETVTAWSGICALD \|  \|  \|  \|  \| \| S.252 \| TVTAWSGICALDGDRT \|  \|  \|  \|  \| \| S.253 \| WSGICALDGDRTFGLV \|  \|  \|  \|  \| \| S.254 \| CALDGDRTFGLVVKDV \|  \|  \|  \|  \| |  |
| --- | --- | --- | --- | --- | --- | --- | --- | --- | --- | --- | --- | --- | --- | --- | --- | --- | --- | --- | --- | --- | --- | --- | --- | --- | --- | --- | --- | --- | --- | --- | --- | --- | --- | --- | --- | --- | --- | --- | --- | --- | --- | --- | --- | --- | --- | --- | --- | --- | --- | --- | --- | --- | --- | --- | --- | --- | --- | --- | --- | --- | --- | --- | --- | --- | --- | --- | --- | --- | --- | --- | --- | --- | --- | --- | --- | --- | --- | --- | --- | --- | --- | --- | --- | --- | --- | --- | --- | --- | --- | --- | --- | --- | --- | --- | --- | --- | --- | --- | --- | --- | --- | --- | --- | --- | --- | --- | --- | --- | --- | --- | --- | --- | --- | --- | --- | --- | --- | --- | --- | --- | --- | --- | --- | --- | --- | --- | --- | --- | --- | --- | --- | --- | --- | --- | --- | --- | --- | --- | --- | --- | --- | --- | --- | --- | --- | --- | --- | --- | --- | --- | --- | --- | --- | --- | --- | --- | --- | --- | --- | --- | --- | --- | --- | --- | --- | --- | --- | --- | --- | --- | --- | --- | --- | --- | --- | --- | --- | --- | --- | --- | --- | --- | --- | --- | --- | --- | --- | --- | --- | --- | --- | --- | --- | --- | --- | --- | --- | --- | --- | --- | --- | --- | --- | --- | --- | --- | --- | --- | --- | --- | --- | --- | --- | --- | --- | --- | --- | --- | --- | --- | --- | --- | --- | --- | --- | --- | --- | --- | --- | --- | --- | --- | --- | --- | --- | --- | --- | --- | --- | --- | --- | --- | --- | --- | --- | --- | --- | --- | --- | --- | --- | --- | --- | --- | --- | --- | --- | --- | --- | --- | --- | --- | --- | --- | --- | --- | --- | --- | --- | --- | --- | --- | --- | --- | --- | --- | --- | --- | --- | --- | --- | --- | --- | --- | --- | --- | --- | --- | --- | --- | --- | --- | --- | --- | --- | --- | --- | --- | --- | --- | --- | --- | --- | --- | --- | --- | --- | --- | --- | --- | --- | --- | --- | --- | --- | --- | --- | --- | --- | --- | --- | --- | --- | --- | --- | --- | --- | --- | --- | --- | --- | --- | --- | --- | --- | --- | --- | --- | --- | --- | --- | --- | --- | --- | --- | --- | --- | --- | --- | --- | --- | --- | --- | --- | --- | --- | --- | --- | --- | --- | --- | --- | --- | --- | --- | --- | --- | --- | --- | --- | --- | --- | --- | --- | --- | --- | --- | --- | --- | --- | --- | --- | --- | --- | --- | --- | --- | --- | --- | --- | --- | --- | --- | --- | --- | --- | --- | --- | --- | --- | --- | --- | --- | --- | --- | --- | --- | --- | --- | --- | --- | --- | --- | --- | --- | --- | --- | --- | --- | --- | --- | --- | --- | --- | --- | --- | --- | --- | --- | --- | --- | --- | --- | --- | --- | --- | --- | --- | --- | --- | --- | --- | --- | --- | --- | --- | --- | --- | --- | --- | --- | --- | --- | --- | --- | --- | --- | --- | --- | --- | --- | --- | --- | --- | --- | --- | --- | --- | --- | --- | --- | --- | --- | --- | --- | --- | --- | --- | --- | --- | --- | --- | --- | --- | --- | --- | --- | --- | --- | --- | --- | --- | --- | --- | --- | --- | --- | --- | --- | --- | --- | --- | --- | --- | --- | --- | --- | --- | --- | --- | --- | --- | --- | --- | --- | --- | --- | --- | --- | --- | --- | --- | --- | --- | --- | --- | --- | --- | --- | --- | --- | --- | --- | --- | --- | --- | --- | --- | --- | --- | --- | --- | --- | --- | --- | --- | --- | --- | --- | --- | --- | --- | --- | --- | --- | --- | --- | --- | --- | --- | --- | --- | --- | --- | --- | --- | --- | --- | --- | --- | --- | --- | --- | --- | --- | --- | --- | --- | --- | --- | --- | --- | --- | --- | --- | --- | --- | --- | --- | --- | --- | --- | --- | --- | --- | --- | --- | --- | --- | --- | --- | --- | --- | --- | --- | --- | --- | --- | --- | --- | --- | --- | --- | --- | --- | --- | --- | --- | --- | --- | --- | --- | --- | --- | --- | --- | --- | --- | --- | --- | --- | --- | --- | --- | --- | --- | --- | --- | --- | --- | --- | --- | --- | --- | --- | --- | --- | --- | --- | --- | --- | --- | --- | --- | --- | --- | --- | --- | --- | --- | --- | --- | --- | --- | --- | --- | --- | --- | --- | --- | --- | --- | --- | --- | --- | --- | --- | --- | --- | --- | --- | --- | --- | --- | --- | --- | --- | --- | --- | --- | --- | --- | --- | --- | --- | --- | --- | --- | --- | --- | --- | --- | --- | --- | --- | --- | --- | --- | --- | --- | --- | --- | --- | --- | --- | --- | --- | --- | --- | --- | --- | --- | --- | --- | --- | --- | --- | --- | --- | --- | --- | --- | --- | --- | --- | --- | --- | --- | --- | --- | --- | --- | --- | --- | --- | --- | --- | --- | --- | --- | --- | --- | --- | --- | --- | --- | --- | --- | --- | --- | --- | --- | --- | --- | --- | --- | --- | --- | --- | --- | --- | --- | --- | --- | --- | --- | --- | --- | --- | --- | --- | --- | --- | --- | --- | --- | --- | --- | --- | --- | --- | --- | --- | --- | --- | --- | --- | --- | --- | --- | --- | --- | --- | --- | --- | --- | --- | --- | --- | --- | --- | --- | --- | --- | --- | --- | --- | --- | --- | --- | --- | --- | --- | --- | --- | --- | --- | --- | --- | --- | --- | --- | --- | --- | --- | --- | --- | --- | --- | --- | --- | --- | --- | --- | --- | --- | --- | --- | --- | --- | --- | --- | --- | --- | --- | --- | --- | --- | --- | --- | --- | --- | --- | --- | --- | --- | --- | --- | --- | --- | --- | --- | --- | --- | --- | --- | --- | --- | --- | --- | --- | --- | --- | --- | --- | --- | --- | --- | --- | --- | --- | --- | --- | --- | --- | --- | --- | --- | --- | --- | --- | --- | --- | --- | --- |

**Supplementary Table 3. Peptide pools for the N protein**

| N.1 | MANQGQRVSWGDESTK |  | N.64 | RSSSANFGDSDLVANG |
| --- | --- | --- | --- | --- |
| N.2 | GQRVSWGDESTKIRGR |  | N.65 | ANFGDSDLVANGSSAK |
| N.3 | SWGDESTKIRGRSNSR |  | N.66 | DSDLVANGSSAKHYPQ |
| N.4 | ESTKIRGRSNSRGRKI |  | N.67 | VANGSSAKHYPQLAEC |
| N.5 | IRGRSNSRGRKINNIP |  | N.68 | SSAKHYPQLAECVPSV |
| N.6 | SNSRGRKINNIPLSFF |  | N.69 | HYPQLAECVPSVSSIL |
| N.7 | GRKINNIPLSFFNPIT |  | N.70 | LAECVPSVSSILFGSY |
| N.8 | NNIPLSFFNPITLQQG |  | N.71 | VPSVSSILFGSYWTSK |
| N.9 | LSFFNPITLQQGAKFW |  | N.72 | SSILFGSYWTSKEDGD |
| N.10 | NPITLQQGAKFWNSCP |  | N.73 | FGSYWTSKEDGDQIEV |
| N.11 | LQQGAKFWNSCPRDFV |  | N.74 | WTSKEDGDQIEVTFTH |
| N.12 | AKFWNSCPRDFVPKGI |  | N.75 | EDGDQIEVTFTHKYHL |
| N.13 | NSCPRDFVPKGIGNRD |  | N.76 | QIEVTFTHKYHLPKDH |
| N.14 | RDFVPKGIGNRDQQIG |  | N.77 | TFTHKYHLPKDHPKTE |
| N.15 | PKGIGNRDQQIGYWNR |  | N.78 | KYHLPKDHPKTEQFLQ |
| N.16 | GNRDQQIGYWNRQTRY |  | N.79 | PKDHPKTEQFLQQINA |
| N.17 | QQIGYWNRQTRYRMVK |  | N.80 | PKTEQFLQQINAYSCP |
| N.18 | YWNRQTRYRMVKGQRK |  | N.81 | QFLQQINAYSCPSEVA |
| N.19 | QTRYRMVKGQRKELPE |  | N.82 | QINAYSCPSEVAKEQR |
| N.20 | RMVKGQRKELPERWFF |  | N.83 | YSCPSEVAKEQRKRKS |
| N.21 | GQRKELPERWFFYYLG |  | N.84 | SEVAKEQRKRKSRSKS |
| N.22 | ELPERWFFYYLGTGPH |  | N.85 | KEQRKRKSRSKSAERS |
| N.23 | RWFFYYLGTGPHADAK |  | N.86 | KRKSRSKSAERSEQEV |
| N.24 | YYLGTGPHADAKFKDK |  | N.87 | RSKSAERSEQEVVPDS |
| N.25 | TGPHADAKFKDKLDGV |  | N.88 | AERSEQEVVPDSLIEN |
| N.26 | ADAKFKDKLDGVVWVA |  | N.89 | EQEVVPDSLIENYTDV |
| N.27 | FKDKLDGVVWVAKDGA |  | N.90 | VPDSLIENYTDVFDDT |
| N.28 | LDGVVWVAKDGAMNKP |  | N.91 | LIENYTDVFDDTQVEM |
| N.29 | VWVAKDGAMNKPTTLG |  | N.92 | YTDVFDDTQVEMIDEV |
| N.30 | KDGAMNKPTTLGSRGA |  | N.93 | FDDTQVEMIDEVTN |
| N.31 | MNKPTTLGSRGANNES |  | N.94 | DVFDDTQVEMIDEVTN |
| N.32 | TTLGSRGANNESKALK |  |  |  |
| N.33 | SRGANNESKALKFDGK |  |  |  |
| N.34 | NNESKALKFDGKVPGE |  |  |  |
| N.35 | KALKFDGKVPGEFQLE |  |  |  |
| N.36 | FDGKVPGEFQLEVNQS |  |  |  |
| N.37 | VPGEFQLEVNQSRDNS |  |  |  |
| N.38 | FQLEVNQSRDNSRSRS |  |  |  |
| N.39 | VNQSRDNSRSRSQSRS |  |  |  |
| N.40 | RDNSRSRSQSRSRSRN |  |  |  |
| N.41 | RSRSQSRSRSRNRSQS |  |  |  |
| N.42 | QSRSRSRNRSQSRGRQ |  |  |  |
| N.43 | RSRNRSQSRGRQQSNN |  |  |  |
| N.44 | RSQSRGRQQSNNKKDD |  |  |  |
| N.45 | RGRQQSNNKKDDSVEQ |  |  |  |
| N.46 | QSNNKKDDSVEQAVLA |  |  |  |
| N.47 | KKDDSVEQAVLAALKK |  |  |  |
| N.48 | SVEQAVLAALKKLGVY |  |  |  |
| N.49 | AVLAALKKLGVYTEKQ |  |  |  |
| N.50 | ALKKLGVYTEKQQQRS |  |  |  |
| N.51 | LGVYTEKQQQRSRSKS |  |  |  |
| N.52 | TEKQQQRSRSKSKERS |  |  |  |
| N.53 | QQRSRSKSKERSNSKT |  |  |  |
| N.54 | RSKSKERSNSKTRDTT |  |  |  |
| N.55 | KERSNSKTRDTTPKNE |  |  |  |
| N.56 | NSKTRDTTPKNENKHT |  |  |  |
| N.57 | RDTTPKNENKHTWKRT |  |  |  |
| N.58 | PKNENKHTWKRTAGKG |  |  |  |
| N.59 | NKHTWKRTAGKGDVTR |  |  |  |
| N.60 | WKRTAGKGDVTRFYGA |  |  |  |
| N.61 | AGKGDVTRFYGARSSS |  |  |  |
| N.62 | DVTRFYGARSSSANFG |  |  |  |
| N.63 | FYGARSSSANFGDSDL |  |  |  |

**Supplementary Table 4. Antibodies used for the intracellular cytokine staining**

| **Antigen** | **Clone** | **Isotype** | **Fluorochrome** | **Source of primary Ab** | **Details of secondary Ab** |
| --- | --- | --- | --- | --- | --- |
| **CD4** | 74-12-4 | IgG2b | PerCP-Cy5.5 | BD Biosciences |  |
| **CD8b** | PPT23 | IgG1 | FITC | Bio-Rad Laboratories |  |
| **TNF** | MAb11 | IgG1 | BV421 | BioLegend |  |
| **IFNγ** | P2G10 | IgG1 | APC | BD Biosciences |  |
| **IL-2** | A150D 3F1 2H2 | IgG2a | PE-Cy7 | ThermoFisher | rat-anti-mouse, IgG2a,  BioLegend |
